# A UL26-PIAS1 complex antagonizes anti-viral gene expression during Human Cytomegalovirus infection

**DOI:** 10.1371/journal.ppat.1012058

**Published:** 2024-05-20

**Authors:** Jessica Ciesla, Kai-Lieh Huang, Eric J. Wagner, Joshua Munger

**Affiliations:** Department of Biochemistry and Biophysics, University of Rochester School of Medicine and Dentistry, Rochester, New York, United States of America; State University of New York Upstate Medical University, UNITED STATES

## Abstract

Viral disruption of innate immune signaling is a critical determinant of productive infection. The Human Cytomegalovirus (HCMV) UL26 protein prevents anti-viral gene expression during infection, yet the mechanisms involved are unclear. We used TurboID-driven proximity proteomics to identify putative UL26 interacting proteins during infection to address this issue. We find that UL26 forms a complex with several immuno-regulatory proteins, including several STAT family members and various PIAS proteins, a family of E3 SUMO ligases. Our results indicate that UL26 prevents STAT phosphorylation during infection and antagonizes transcriptional activation induced by either interferon α (IFNA) or tumor necrosis factor α (TNFα). Additionally, we find that the inactivation of PIAS1 sensitizes cells to inflammatory stimulation, resulting in an anti-viral transcriptional environment similar to ΔUL26 infection. Further, PIAS1 is important for HCMV cell-to-cell spread, which depends on the presence of UL26, suggesting that the UL26-PIAS1 interaction is vital for modulating intrinsic anti-viral defense.

## Author summary

Cells possess defense mechanisms to limit viral infection and viruses encode countermeasures to block these cellular defenses to ensure viral spread. These virus-host interactions frequently determine the outcome of infection. Our data indicate that a gene encoded by Human Cytomegalovirus (HCMV), UL26, interacts with several cellular proteins critical to host defense. Consistent with this finding, we find that UL26 blocks several different anti-viral defense pathways. One of the cellular proteins that interacts with UL26, PIAS1, can also regulate several anti-viral pathways, as it normally functions to curtail inflammatory gene expression. We find that PIAS1’s anti-inflammatory activity is utilized during HCMV infection to block anti-viral gene expression and support productive infection. Our data highlight a novel host-pathogen molecular interaction that can influence the outcome of HCMV infection, which is an important consideration given that HCMV induces significant morbidity in neonates and the immunosuppressed.

## Introduction

Human Cytomegalovirus (HCMV) is a widespread pathogen belonging to the *Herpesviridae* family that causes severe morbidity during congenital infection and in immunosuppressed individuals. In the United States, 1 in 200 children are born with HCMV, and of this population, approximately 10% are born with symptoms including blindness, deafness, microcephaly, jaundice, seizures, and, in the most severe cases, death [1]. Other reports suggest up to 20–25% of HCMV-positive newborns are symptomatic at birth or will develop long-term medical complications [2]. Currently, there are no FDA-approved vaccines for HCMV, and anti-viral therapeutic options exhibit toxicity, low bioavailability, and the emergence of drug-resistant strains in immunosuppressed populations [3–5]. Understanding the determinants of successful HCMV infection could provide novel points of therapeutic intervention to lessen the disease burden of HCMV.

The host’s innate immune response is critical for limiting viral infection. Activation of innate immune pathways, including nuclear factor kappa-light-chain-enhancer of activated B cells (NFκB) [6–8] and Janus kinase/signal transducer and activator of transcription (JAK/STAT) [9,10] promote anti-viral gene expression to restrict viral infection. Viruses have evolved mechanisms to evade detection or block innate immune activation to replicate efficiently. With respect to HCMV, the virion tegument contains a matrix of proteins between the nucleocapsid and envelope that are released into the cytoplasm immediately upon infection. Many tegument proteins antagonize various cellular anti-viral activities. The most abundant tegument protein, pp65, blocks IRF3 activation to restrict type I interferon signaling [11]. The pp71 protein binds the innate immune effector Daxx to prevent Daxx-mediated silencing of the HCMV Major Immediate Early Promoter (MIEP) [12]. The UL23 protein restricts Type II interferon gene expression during infection by interacting with the human n-Myc interactor [13]. The UL26 tegument protein, which is important for high titer replication [14,15], restricts the expression of cytokine signaling genes during infection [16] and antagonizes TNFα-induced NFκB activation [17]. However, the mechanisms by which UL26 modulates host innate immune signaling and supports high titer viral replication are unclear.

Here, we explored the UL26 interactome during early-stage infection using proximity-based proteomics [18]. To identify UL26-associated proteins that likely contribute to high titer viral replication, we compared the interactome of wildtype UL26 with that of a UL26 C-terminal deletion mutant (UL26ΔC) that phenocopies UL26-deletion viral growth kinetics [19]. Our data suggest that the full-length UL26 protein, but not UL26ΔC, interacts with PIAS1 (protein inhibitor of activated STAT 1), a key modulator of STAT and NFκB transcription factors [20]. PIAS proteins negatively regulate anti-viral gene expression by binding DNA at specific promoter regions and antagonizing transcription factor binding [21,22]. PIAS proteins also act as E3-SUMO ligases and conjugate a small ubiquitin-modifier (SUMO) polypeptide to modulate protein activity, including inhibition of transcription factor activity [23]. Our results indicate that cells lacking PIAS1 are less permissive to HCMV spread and have increased expression of anti-viral genes, similar to what is observed during ΔUL26 infection.

## Results

### The proximal UL26 interactome contains several host-defense regulatory proteins

**Fig 1:**
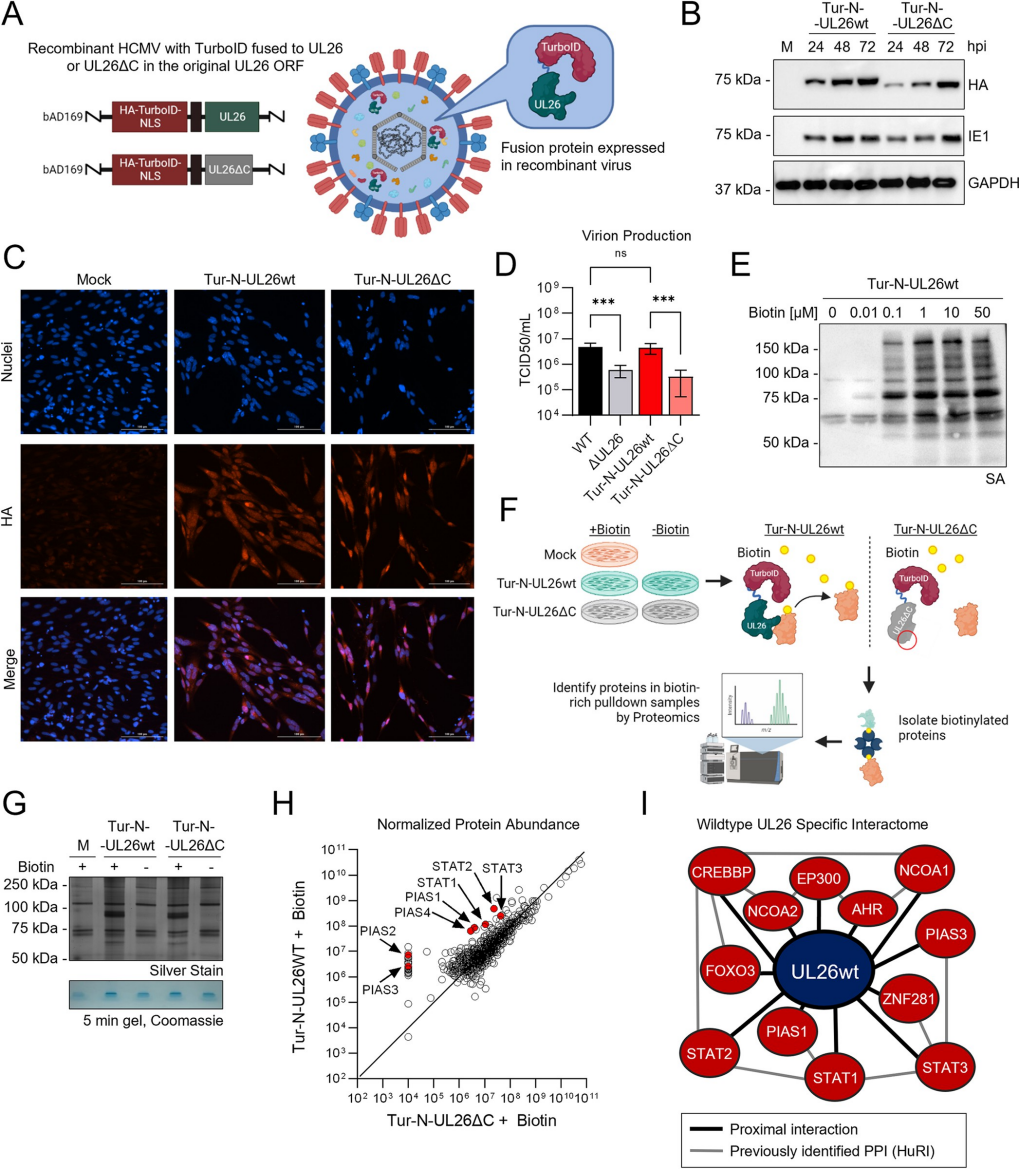
The proximal UL26 interactome contains several host-defense regulatory proteins. (**A**) Recombinant HCMV containing HA-TurboID fused to the N-terminus of wildtype UL26 (Tur-N-UL26wt) or UL26ΔC (Tur-N-UL26ΔC) (**B**) Western blot showing the expression of HA-TurboID fused to UL26wt or UL26ΔC in MRC5s infected with Tur-N-UL26wt or Tur-N-UL26ΔC (MOI = 3) at the indicated hour post infection (hpi) compared to mock (M). (**C**) Immunofluorescence images of MRC5s infected with Tur-N-UL26wt or Tur-N-UL26ΔC (MOI = 3) at 48 hpi. Tur-N-UL26wt and Tur-N-UL26ΔC protein detected by anti-HA; nuclei stained with Hoechst. (**D**) Infectious virions produced by MRC5s infected with wild-type HCMV (WT), UL26-deletion (ΔUL26), Tur-N-UL26wt or Tur-N-UL26ΔC at 120 hpi, determined by TCID50 analysis. Virion production is represented as the mean of 6 biological replicates +/- SEM. FDR-adjusted p-values determined using 2-way ANOVA followed by two-stage step-up method of Benjamini, Krieger and Yekutieli n.s = not significant, *p<0.033, **p<0.002, ***p<0.001. (**E**) Western blotting with avidin-peroxidase showing the accumulation of biotinylated protein in MRC5s infected with Tur-N-UL26wt (MOI = 3). Infected cells were incubated with the indicated concentration of biotin at 23 hpi, and protein was harvested at 24 hpi. (**F-I**) TurboID-mediated proximity labeling experiment. (**F**) Experiment overview: cells infected with Mock, Tur-N-UL26wt, or Tur-N-UL26ΔC (MOI = 3) and treated with 1 μM biotin at 23 hpi. Total protein harvested at 24 hpi, biotinylated proteins isolated by affinity purification using streptavidin-bound magnetic beads then identified by LC-MS/MS (n = 1). (**G**) Silver stain of affinity pulldown samples showing total biotin-enriched protein (top). Remaining affinity pulldown sample concentrated on a 5 minute gel and stained with Coomassie for proteomic analysis (bottom). (**H**) Protein abundance data from LC-MS/MS of Tur-N-UL26wt and Tur-N-UL26ΔC affinity pulldown samples median-normalized and represented as a scatter plot. STAT and PIAS proteins highlighted in red. (**I**) Interactome of 12 of the 67 human proteins enriched in Tur-N-UL26wt+Biotin relative to Tur-N-UL26ΔC+Biotin (Fold Change > 5) identified using the Human Reference Protein Interactome Mapping Project (HuRI) database. Black lines indicate wildtype UL26-specific proximal interactions detected in (H), grey lines indicate previously identified protein-protein interactions (PPIs) from HuRI.

To investigate the mechanisms through which UL26 contributes to HCMV replication, we sought to identify proteins that interact with UL26 during infection. Towards this end, we fused UL26 to the TurboID biotin ligase, which conjugates biotin to proximal proteins with faster kinetics relative to previous biotin ligase domains [18]. In addition to TurboID tagging WT UL26, we also tagged a UL26 variant in which the C-terminal 38 amino acids have been deleted (UL26ΔC). Recombinant HCMV strains that express this UL26ΔC variant behave similarly to ΔUL26 strains with respect to their growth kinetics [19]. Given that UL26’s C-terminus is critical for its function [19], we reasoned that the proteins interacting with WT UL26, but not the UL26ΔC mutant, are more likely to be important for UL26’s contributions to HCMV infection. We created recombinant HCMV strains containing TurboID-tagged versions of WT UL26 (Tur-N-UL26wt) or the UL26ΔC mutant (Tur-N-UL26ΔC) in the original UL26 genomic locus (Fig 1A). Both TurboID-fused proteins were stably expressed, accumulated normally during infection (Fig 1B), and localized to the nucleus as expected (Fig 1C). Further, TurboID tagging of WT UL26 did not impact HCMV viral titers, suggesting that UL26 was still functional upon tagging (Fig 1D). Similarly, TurboID tagging of UL26ΔC grew to a similar titer as the ΔUL26 virus (Fig 1D).

We next assessed the biotinylation activity of our TurboID-tagged recombinant viruses as well as the concentration of biotin that would yield robust proximal biotinylation over an hour time frame. Our results indicate pulsing with 1 μM biotin resulted in maximal biotinylation as assessed by streptavidin HRP signal (Fig 1E). Moving forward to the proteomic analysis, fibroblasts were mock infected or infected with Tur-N-UL26wt, or Tur-N-UL26ΔC (MOI = 3) for 23 hours, treated with 1 μM biotin or vehicle for 1 hour, and purified biotinylated proteins were isolated via streptavidin affinity (Fig 1F). The resulting proteins were analyzed by silver stain (Fig 1G). As expected, streptavidin affinity purified more proteins in the infected samples treated with biotin relative to either the non-biotin treated infected cells or the mock-infected biotin treated cells (Fig 1G, top). Purified samples were then concentrated by 5-minute SDS-PAGE, stained for total protein (Fig 1G, bottom), and analyzed by LC-MS/MS to identify the biotinylated proteins.

In total, LC-MS/MS identified 601 Human and HCMV proteins in the streptavidin affinity purified samples (S1 Table). Among those 601 identified proteins, several were preferentially biotinylated by the full-length UL26-TurboID versus UL26ΔC-TurboID (Fig 1H). Specifically, sixty-eight proteins were ≥5-fold enriched with respect to interacting with WT UL26 relative to the UL26ΔC mutant, suggesting the UL26 C-terminus is necessary for their interaction (S2 Table). The UL31 protein was the only viral protein among these 68 proteins and was ~8-fold enriched in the WT UL26 sample relative to the UL26ΔC sample (S3 Table). Twenty additional viral proteins were found to interact with both WT UL26 and the UL26ΔC mutant (S3 Table). Of the 67 human proteins that interacted preferentially with the full-length UL26, many are involved in innate immune signaling, including several STAT and PIAS family members (Fig 1H and S2 Table). Further, several of these proteins have been found to complex together, as predicted by the Human Reference Interactome (HuRI) [24] (Fig 1I). Collectively, these results suggest that UL26 associates with a complex involved in the modulation of host responses to infection, consistent with activities previously linked to UL26 [17,25].

### The UL26 protein blocks STAT activation and cytokine-mediated transcriptional activation

**Fig 2:**
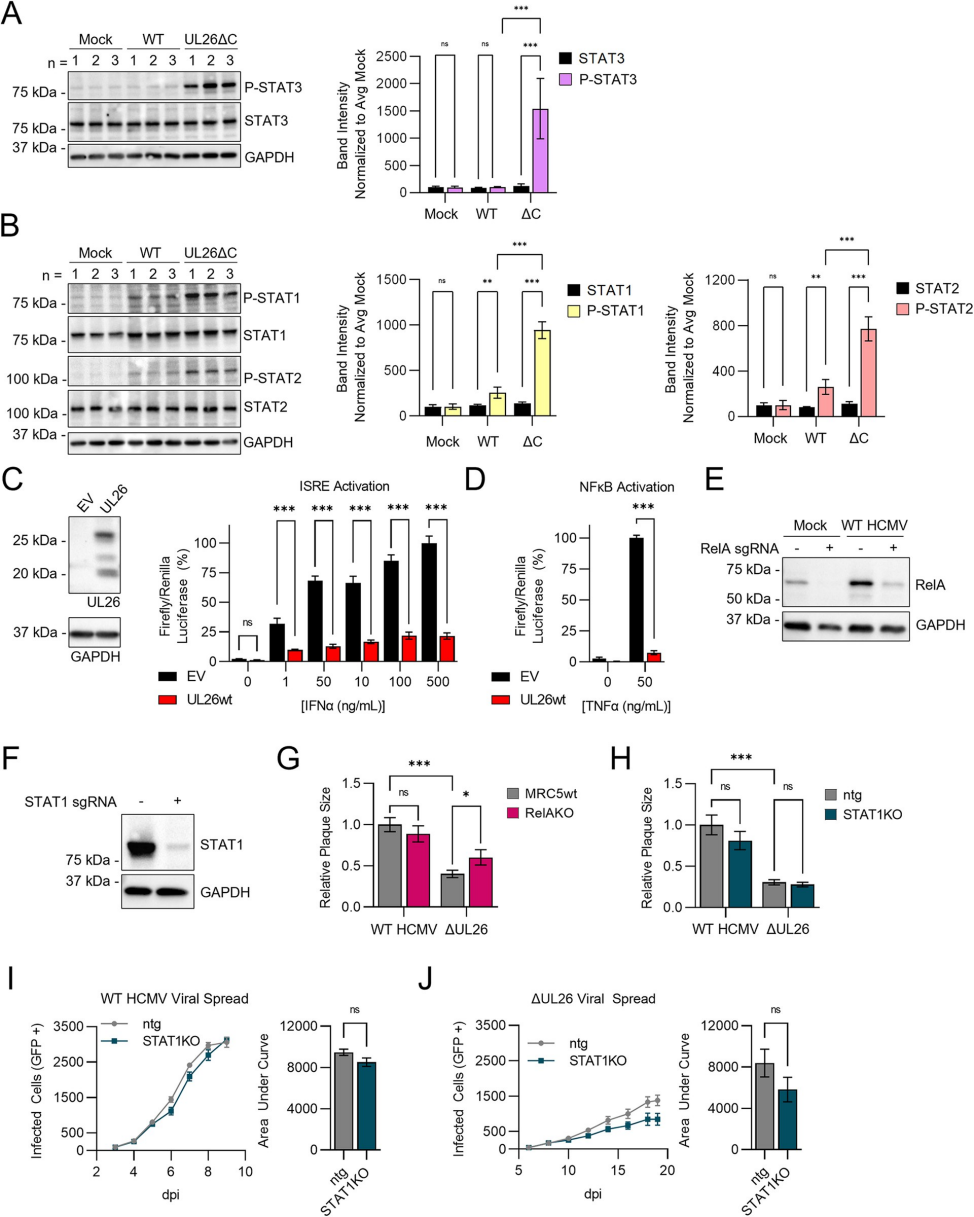
The UL26 protein blocks TNFα and IFNα signaling. (**A**-**B**) MRC5 cells infected with mock, wildtype HCMV (WT), or UL26ΔC mutant virus (MOI = 3). Protein harvested from three independent biological replicates at 0.5 (A) or 24 hpi (B) and processed by western blot as indicated (left). For each replicate, band intensity for the indicated protein was quantified relative to GAPDH and normalized to the average mock-infected band intensity (mock set to 100). Data are represented as the mean value of three biological replicates +/- SD (right). FDR-adjusted p-values determined using 2-way ANOVA followed by two-stage step-up method of Benjamini, Krieger and Yekutieli n.s = not significant, *p<0.033, **p<0.002, ***p<0.001. (**C** and **D**) Dual-Glo luciferase assay in HEK293T cells containing a renilla luciferase plasmid and Interferon Stimulated Response Element (ISRE) firefly luciferase reporter plasmid (D), or NFκB firefly luciferase reporter plasmid (E). UL26 is expressed in HEK293T cells transfected with a UL26 expression plasmid for 48 hr (C, left). HEK293T cells containing Dual-Glo luciferase reporter assay system were transfected with empty vector (EV) or a UL26 expression plasmid for 48 hr, then stimulated with the indicated concentration of IFNα (C) or TNFα (D) for 24 hr. Firefly and renilla luciferin were quantified using a DualGlo luciferase kit. For each sample, firefly luciferin was normalized to renilla luciferin and represented as the mean of 4 biological replicates +/- SE. FDR-adjusted p-values determined using 2-way ANOVA followed by two-stage step-up method of Benjamini, Krieger and Yekutieli n.s = not significant, *p<0.033, **p<0.002, ***p<0.001. (**E**) Immunoblot of protein harvested from MRC5s transfected with CRISPR-Cas9 complexed to RelA-targeted sgRNA then infected with mock or wildtype HCMV. (**F**) Immunoblot of protein harvested from MRC5 cells transfected with CRISPR-Cas9 complexed to STAT1-targeted sgRNA or a non-targeting guide (ntg). (**G** and **H**) Plaque size of wildtype HCMV or ΔUL26 in RelAKO cells compared to parental MRC5s (G) or STAT1KO cells compared to ntg cells (H). The area of 20 representative plaques were quantified for each condition and normalized to the mean plaque size of wildtype HCMV in parental (G) or ntg (H). Mean plaque size for each condition is represented as the mean +/- SE. FDR-adjusted p-values determined using 2-way ANOVA followed by two-stage step-up method of Benjamini, Krieger and Yekutieli n.s = not significant, *p<0.033, **p<0.002, ***p<0.001. (**I** and **J**) Viral spread assay of GFP-expressing wildtype HCMV (I, MOI = 0.01) or GFP-expressing ΔUL26 virus (J, MOI = 0.01) in STAT1KO (teal) cells compared to ntg (grey). Number of infected cells (GFP+) quantified for 6 biological replicates at the indicated day post infection (dpi) and represented as the mean +/- SE. Total area under curve (AUC) calculated for viral spread in each cell line and plot as a bar graph +/- SE (right); p-values determined by student’s unpaired t-test, ns = not significant, *p<0.033, **p<0.002, ***p<0.001.

While UL26 has been found to attenuate NFκB signaling [17], the associated proteins identified above suggest it may also modulate STAT signaling. To explore this possibility, we analyzed STAT phosphorylation in WT HCMV or UL26ΔC infected cells. STAT phosphorylation is a dynamic modification; preliminary testing suggested STAT3 was maximally phosphorylated at early times post infection (0.5 hpi) while STAT1 maximum phosphorylation occurred at 24 hpi (S1 Fig). Phospho-STAT3 levels were significantly increased in UL26ΔC-infected cells relative to mock or WT HCMV infection (Fig 2A). Similarly, phospho-STAT1 and phospho-STAT2 levels were increased in UL26ΔC-infected cells relative to mock or WT infection (Fig 2B). These results suggest that the UL26 C-terminus is necessary to restrict STAT phosphorylation during HCMV infection.

We previously have found that UL26 is sufficient to antagonize TNFα-mediated NFκB activation [17] and is necessary to prevent the induction of interferon-stimulated gene (ISG) expression during infection [16]. Given our findings that STAT phosphorylation is induced in UL26ΔC infection, we assessed whether UL26 is sufficient to inhibit activation of the Interferon Stimulated Response Element (ISRE), a promoter for ISGs induced by activated STAT transcription factor complexes [26]. HEK293T cells were transfected with an ISRE firefly luciferase reporter plasmid and control vector (EV) or UL26-expression vector (Fig 2C, left). Treatment with increasing amounts of IFNα induced ISRE-dependent luciferase activity, which was inhibited by UL26 expression (Fig 2C, right), suggesting that UL26 expression is sufficient to antagonize IFNα-induced ISRE activation. We also analyzed the impact of UL26 expression on TNFα-induced NFκB activation and found that, similar to previous results [17], UL26 expression was sufficient to inhibit TNFα-induced activation of NFκB-dependent luciferase activity (Fig 2D). Together, these data indicate that UL26 is sufficient to antagonize IFNα and TNFα-induced transcriptional activation.

Given that ΔUL26 infection fails to block the expression of cytokine signaling genes [16], we assessed whether the inactivation of STAT1 or RELA, endpoint transcription factors associated with activated STAT and NFκB signaling, respectively, would rescue ΔUL26 replication defects. RelA and STAT1 knockout (KO) MRC5 cell lines were generated through CRISPR-CAS9 gene targeting, resulting in a significant knockdown in expression in polyclonal cell populations relative to non-targeting guide controls (ntg) (Fig 2E and 2F). RELA targeting slightly increased the plaque size associated with ΔUL26 infection, although these plaques were still substantially smaller than WT HCMV in the RELA targeted cells (Fig 2G). In contrast, the absence of STAT1 did not impact ΔUL26 plaque size (Fig 2H). Further, the viral spread of WT HCMV or ΔUL26 was not impacted by the lack of STAT1 (Fig 2I and 2J). These results suggest that inactivation of either RELA or STAT1 alone is not sufficient to rescue the viral spread defects associated with ΔUL26 infection.

### PIAS protein abundance and SUMOylation are enhanced during HCMV infection

**Fig 3:**
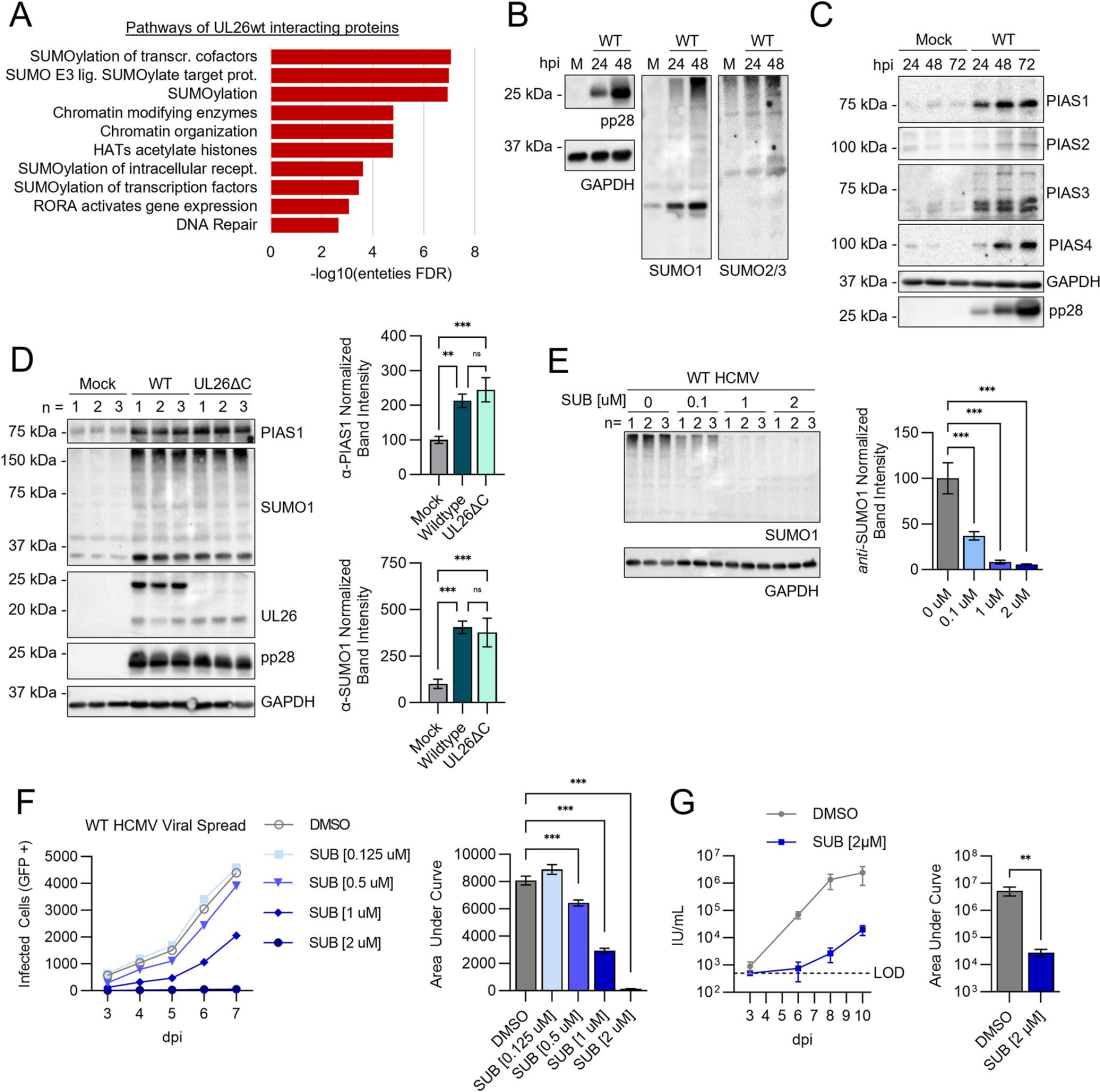
HCMV induces the expression of PIAS family E3 SUMO ligases and increases protein SUMOylation, which is important for HCMV spread. (**A**) Reactome analysis of 67 human proteins enriched in Tur-N-UL26wt+Biotin relative to Tur-N-UL26ΔC+Biotin (Fold Change > 5). Reactome pathways with the top 10 entities FDR represented as -log10(entities FDR). (**B** and **C**) MRC5 cells infected with mock (M) or wildtype HCMV (WT, MOI = 3). At the indicated hour post infection (hpi), protein harvested for western blot analysis. (**D**) MRC5 cells infected with mock, WT, or UL26ΔC mutant virus (MOI = 3). Protein harvested at 48 hpi and processed by immunoblot as indicated. Three independent replicates were carried out for each sample group (n = 1–3). For each replicate, band intensity for PIAS1 or SUMO1 was quantified relative to the GAPDH loading control and normalized to the average mock-infected band intensity (100). The mean value of three biological replicates +/- SD was plotted as bar graphs for PIAS1 and SUMO1 (right). FDR-adjusted p-values determined using 2-way ANOVA followed by two-stage step-up method of Benjamini, Krieger and Yekutieli n.s = not significant, *p<0.033, **p<0.002, ***p<0.001. (**E**) MRC5 cells infected with WT (MOI = 3) and treated with the indicated concentration of Subasumstat (SUB). At 48 hpi, protein harvested and analyzed by western blot (left). Three independent replicates were carried out for each sample group (n = 1–3). For each replicate, band intensity for SUMO1 was quantified relative to the GAPDH loading control and normalized to the average mock-infected band intensity (100). The mean value of three biological replicates +/- SD was plotted as bar graphs (right). FDR-adjusted p-values determined using 2-way ANOVA followed by two-stage step-up method of Benjamini, Krieger and Yekutieli n.s = not significant, *p<0.033, **p<0.002, ***p<0.001. (**F**) Viral spread assay of GFP-expressing wildtype HCMV in MRC5 cells treated with the indicated concentration of Subasumstat (SUB) during infection. Number of infected cells (GFP +) quantified for 6 biological replicates at the indicated day post infection (dpi) and represented as the mean +/- SE in an XY-plot for each day (left). Total area under curve (AUC) calculated for viral spread in each cell line and plot as a bar graph +/- SE (right); FDR-adjusted p-values determined using 2-way ANOVA followed by two-stage step-up method of Benjamini, Krieger and Yekutieli n.s = not significant, *p<0.033, **p<0.002, ***p<0.001. (**G**) Viral growth assay of MRC5 cells treated with Subasumstat (2 μM) or DMSO during infection with GFP-expressing WT HCMV (MOI = 0.01). Medium was harvested from cells infected at the indicated dpi. Infectious units (IU) per mL was quantified for each sample and plotted as the mean +/- SEM (left) of 12 biological replicates. Total area under the curve for each treatment was calculated and plotted as a bar graph +/+- SEM (right). p-values determined by student’s unpaired t-test, ns = not significant, *p<0.033, **p<0.002, ***p<0.001.

To gain further clues about the possible activities of the UL26-proximal complex identified above, we performed an ontology analysis of the 67 Human proteins enriched in the WT UL26 versus UL26ΔC samples (S4 Table) [24]. Our analysis revealed that these proteins were preferentially associated with various aspects of protein SUMOylation (Fig 3A). SUMO proteins are small (~10 kDa) protein modifiers that are covalently attached to target proteins by E3-SUMO ligases, including Protein Inhibitor of Activated STAT (PIAS) proteins, in a process known as SUMOylation. SUMOylation is a reversible post-translational modification that controls diverse molecular functions, including chromatin structure, DNA repair, transcription, cell cycle progression, and the innate immune response [27]. SUMOylation supports the activation of intrinsic immunity, and SUMOylation of some viral proteins restricts viral replication, yet many viruses have evolved mechanisms to modulate SUMOylation and counter this anti-viral activity [28–30]. For HCMV, SUMO over-expression has been found to enhance HCMV replication [31], suggesting that it might benefit infection. Consistent with these findings, we observe protein SUMOylation is enhanced during WT HCMV infection (Fig 3B). WT HCMV infection also substantially increased the accumulation of PIAS-type E3-SUMO ligases, including PIAS1, PIAS3, and PIAS4, as well as PIAS2, albeit to a much lesser extent (Fig 3C). Similar results were observed during UL26ΔC infection, SUMO1 and PIAS1 levels were increased (Fig 3D). To determine if SUMOylation is broadly important for HCMV replication, we treated cells with the SUMOylation inhibitor Subasumstat [32]. Subasumstat treatment did not impact cell viability (S2 Fig), but caused a dose-dependent decrease in HCMV-induced SUMOylation and inhibited viral spread (Fig 3E and 3F). Further, Subasumstat treatment attenuated the accumulation of infectious virions by ~100-fold (Fig 3G), indicating that SUMOylation is important for HCMV infection.

### PIAS1 inactivation potentiates anti-viral gene expression

**Fig 4:**
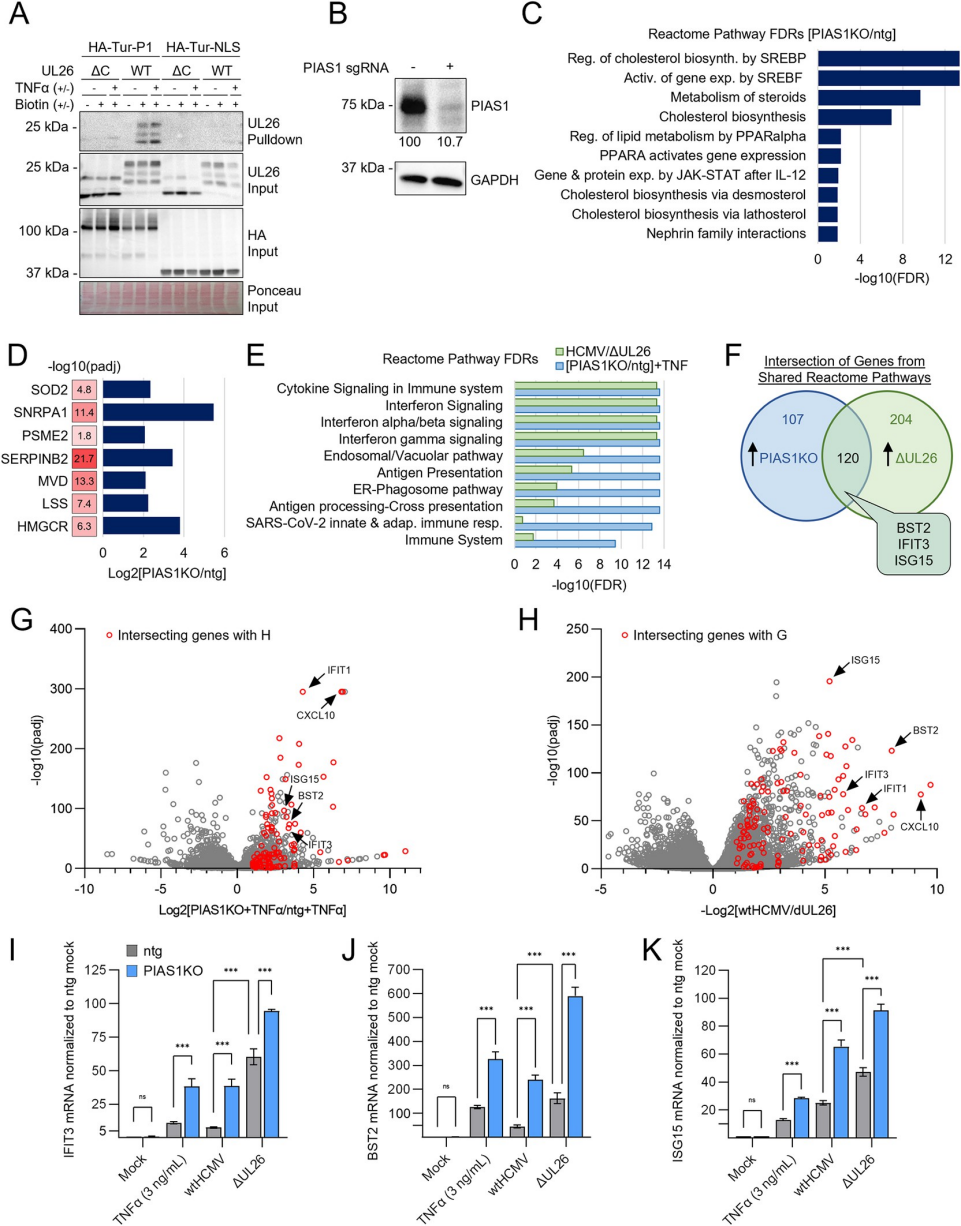
PIAS1 inactivation sensitizes cells to inflammatory stimulation resulting in increased anti-viral gene expression that phenocopies ΔUL26 infection. (**A**) HEK293T cells transfected with HA-TurboID-PIAS1 (HA-Tur-P1), HA-TurboID-NLS (HA-Tur-NLS), UL26 WT, or UL26ΔC, as indicated. At 47 hours post transfection, cells were treated with biotin (1 μM). Protein harvested from cells at 48 hours post transfection (1 hr biotin incubation). Biotinylated proteins extracted by streptavidin affinity purification. Input and pulldown samples processed by Western blot as indicated. (**B**) Western blot of protein harvested from MRC5 cells transfected with CRISPR-cas9 RNP complexed to sgRNAs towards PIAS1 or a non-targeting guide (ntg) RNA. Band intensity of PIAS1 normalized to GAPDH for each sample, the ntg PIAS1 band intensity was standardized to 100. (**C**-**G**) Ntg and PIAS1 knockout (KO) cells treated with vehicle or TNFα (3 ng/mL) for 24 hr. RNA harvested and analyzed by RNA-seq (n = 3). (**C**) Reactome analysis of genes enriched in vehicle-treated PIAS1KO cells relative to ntg (Log2[PIAS1KO/ntg]> 2]. Reactome pathways with the top 10 entities FDR represented as -log10(entities FDR). (**D**) Log2 fold change enrichment in PIAS1KO cells relative to ntg and padj values for individual genes significantly enriched in vehicle-treated PIAS1KO cells relative to ntg. (**E**, blue) Reactome analysis of genes enriched in TNFα-treated PIAS1KO cells relative to ntg. Reactome pathways with the top 10 entities FDR represented as -log10(entities FDR). (E and F, green; **H**) Data adapted from GEO #GSE137065. MRC5 cells infected with WT or ΔUL26 HCMV at MOI = 3 for 24 hr. RNA harvested and analyzed by RNA-seq. (E, green) Reactome pathways of genes enriched in cells infected with ΔUL26 relative to WT that overlap with PIAS1KO RNA seq experiment. Entities FDR is represented as -log10(entities FDR). (**F**) Venn diagram of entities from reactome analysis top 10 pathways represented in E. Genes enriched in TNFα-treated PIAS1KO cells relative to ntg cells (blue, 227 genes) and ΔUL26 relative to WT (green, 324 genes). (**G** and **H**) Volcano plot of RNA-seq data from TNFα-treated PIAS1KO and ntg cells (G). RNA-seq data from cells infected with WT or ΔUL26 (H). 120 intersecting genes shown in (F) are represented in red. (**I**—**K**) RTqPCR quantification of IFIT3 (I) BST2 (J) and ISG15 (K) in ntg and PIAS1KO cells treat with mock, wtHCMV (MOI = 3), ΔUL26 (MOI = 3) or TNFα (3 ng/mL) for 24 hr. Data represented as the mean of 3 biological replicates +/- SD; FDR-adjusted p-values determined using 2-way ANOVA followed by two-stage step-up method of Benjamini, Krieger and Yekutieli n.s = not significant, *p<0.05, **p<0.01, ***p<0.001.

Our proteomics data indicate that WT UL26 preferentially biotinylated several PIAS family members relative to the UL26ΔC mutant. While each of the different PIAS family members could play important roles during HCMV infection, we focused on PIAS1 as it abundantly accumulated at early times post-infection (Fig 3C). To further examine this interaction, we wanted to test if a TurboID-tagged PIAS1 would biotinylate UL26, i.e., the reverse of the experiments in Fig 1. Additionally, we wanted to determine if the proximal interaction occurred in the absence of other viral proteins present during infection. HA-TurboID-PIAS transfected cells produced a protein at the expected 100 kDa size (S3A Fig) and displayed a similar biotinylation efficiency as TurboID-transfected control cells (S3B Fig). Co-transfection with WT UL26 or UL26ΔC with subsequent purification of biotinylated proteins indicated that TurboID-tagged PIAS1 biotinylated full-length UL26 but not the UL26 C-term deletion mutant, consistent with our mass spectrometry results (Figs 4A and S4). This proximal interaction occurred to a similar extent regardless of whether cells were treated with cytokines (Fig 4A), which we reasoned might potentially stimulate the UL26-PIAS1 interaction. These results suggest that UL26 and PIAS1 proximally interact regardless of viral infection.

Similar to UL26, PIAS1 has been found to modulate multiple innate immune signaling pathways, including SUMOylating STAT1’s C-terminal region, attenuating STAT1’s DNA-binding activity, and inhibiting inflammatory gene expression [33]. Additionally, PIAS1 SUMOylates NFκB signaling components, e.g., inhibiting anti-viral gene expression through SUMOylation of IKKα and binding RELA [34,35]. To examine PIAS1’s anti-viral activity, we targeted it via the delivery of CRISPR-ribonucleoprotein (RNP) complexes, which substantially reduced PIAS1 protein accumulation in polyclonal cell populations (Fig 4B), but did not substantially alter the rate of cellular proliferation relative to the non-targeting guide (ntg) control cells (S5 Fig). PIAS1 inactivation did not impact the total levels of SUMOylation observed during infection (S6 Fig), indicating that PIAS1 is not necessary for the global induction of SUMOylation induced by HCMV.

Given PIAS1’s described role in inhibiting inflammatory gene expression [33], and the possibility that it is important to HCMV infection, we performed RNA-seq analysis on control versus PIAS1-targeted cells. This analysis revealed 302 genes significantly more abundant in cells lacking PIAS1 (PIAS1KO) relative to the non-targeting guide (ntg) control cell line (S5 Table). Ontology analysis of these 302 genes revealed that PIAS1 inactivation induced genes involved in cholesterol metabolism (Fig 4C and S5 Table), including 3-hydroxy-3-methylglutaryl-CoA reductase (HMGCR), lanosterol synthase (LSS), and mevalonate diphosphate decarboxylase (MVD) (Fig 4D and S5 Table). These findings are consistent with previous reports indicating that PIAS1 suppresses the expression of cholesterol-associated enzymes [36,37]. In addition to cholesterol metabolism, PIAS1 inactivation increased the expression of genes associated with IL12-induced JAK-STAT signaling (Fig 4C and S5 Table).

Given that PIAS1 modulates NFκB signaling [34,35], we tested whether inactivation of PIAS1 impacted the response to TNFα treatment, an activator of NFκB signaling. TNFα-treated PIAS1KO cells exhibited substantially larger increases in genes involved in cytokine signaling and the innate immune response relative to TNFα-treated control cells (Fig 4E and S6 and S7 Tables). Notably, similar pathways are induced when comparing ΔUL26 infection relative to WT HCMV infection (Fig 4E and S8 Table, ΔUL26 data from [25], GEO #GSE137065). Of the genes within these intersecting pathways, 120 were significantly induced in both ΔUL26 infection and TNFα-treated PIAS1 KO cells relative to their respective control conditions (Fig 4F and S9 Table). Many of these 120 overlapping genes are interferon-stimulated genes (ISGs) including IFIT1-3, ISG15, CXCL10, and BST2. These genes were among the most strongly induced in both conditions (Fig 4G aand 4H, red). Collectively, these results suggest that the transcriptional environment induced by ΔUL26 infection has similarities to TNFα-treated PIAS1KO cells.

Given that PIAS1KO cells were sensitized to TNFα treatment, an inflammatory stimulus, we hypothesized that PIAS1KO cells may be more sensitive to the pro-inflammatory signaling associated with HCMV infection. To examine this possibility, we analyzed the expression of three ISGs identified above, IFIT3, BST2, and ISG15. Consistent with our previous results, there was no significant difference in the expression of these genes in either cell line under non-treated conditions (Fig 4I–4K). In contrast, either TNFα-treatment or ΔUL26 infection of PIAS1KO cells strongly induced the expression of these ISGs relative to control cells (Fig 4I–4K). In addition, the inactivation of PIAS1 also resulted in the strong induction of ISG expression during WT HCMV infection (Fig 4I–4K), suggesting that PIAS1 is important in limiting ISG expression during normal HCMV infection. During ΔUL26 infection, the expression of these ISGs was elevated, but to a lesser extent (Fig 4I–4K), suggesting that UL26 is necessary for PIAS1 to fully suppress ISG gene expression during infection. Taken together, these experiments indicate that inactivation of PIAS1 potentiates anti-viral transcriptional responses resulting in the strong induction of cytokine-signaling genes upon HCMV infection.

### PIAS1 inactivation sensitizes cells to cytokine treatment to limit viral infection

**Fig 5:**
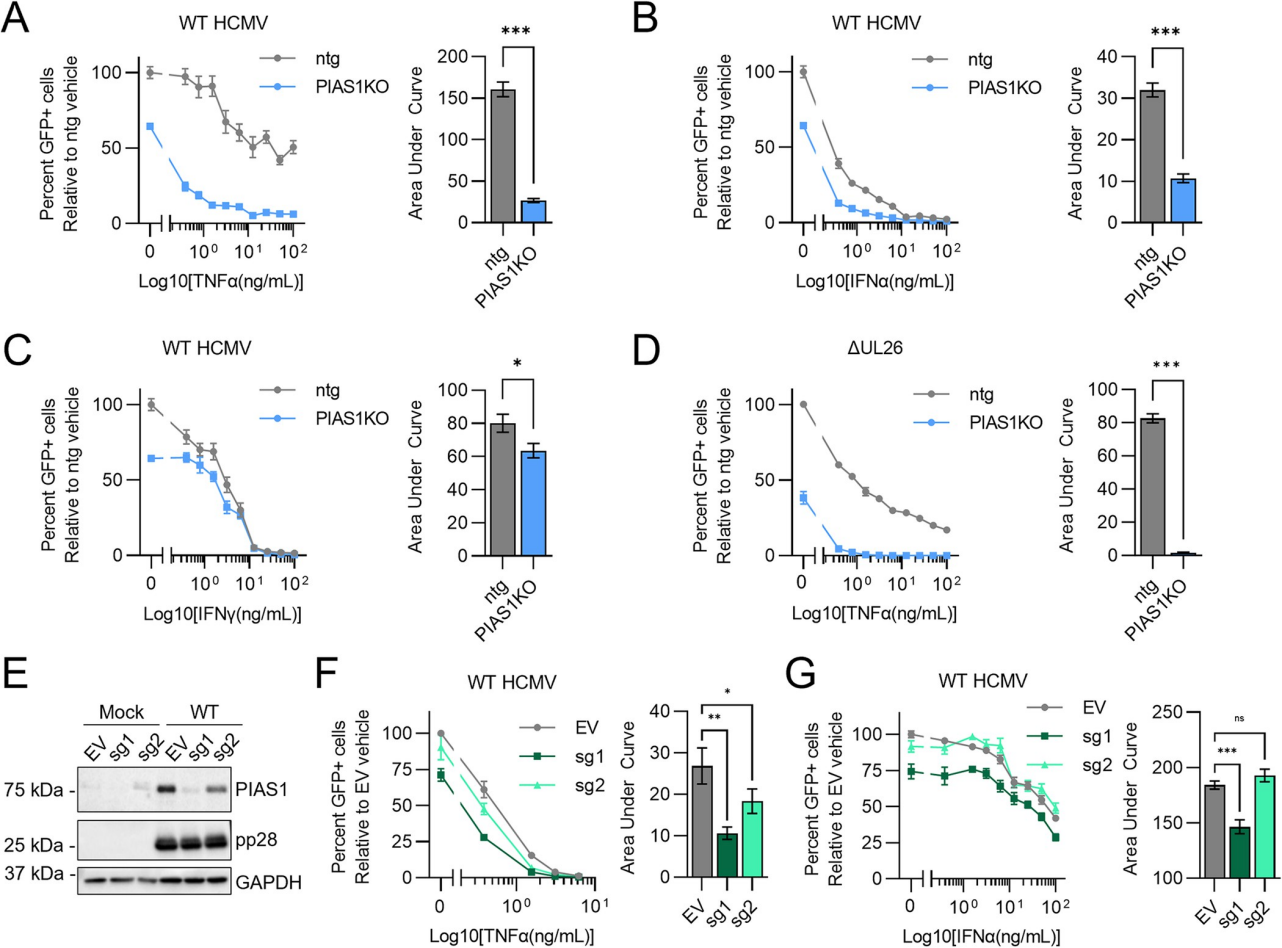
PIAS1 inactivation sensitizes cells to anti-viral cytokine treatment. (**A—C**) Viral spread assay. Ntg (grey) and PIAS1KO (light blue) cells pre-treated with control medium or a 9-point dose curve of TNFα (A) IFNα (B) IFNγ (C) for 24 hr then infected with GFP-expressing wildtype HCMV (WT HCMV, MOI = 0.05). At 6 days post infection (dpi), total number of infected (GFP+) cells were quantified for each condition and normalized to control-treated ntg (100%). (**D**) Viral spread assay. Ntg (grey) and PIAS1KO (light blue) cells pre-treated with control medium or a 9-point dose curve of TNFα for 24 hr then infected with GFP-expressing ΔUL26 (MOI = 0.1). At 7 days post infection (dpi), total number of infected (GFP+) cells were quantified for each condition and normalized to control-treated ntg (100%). (**E-G**) MRC5 cells transduced with CRISPRi plasmid containing empty vector (EV, grey) or PIAS1 sgRNAs (sg1, dark green & sg2, light green) constructs. Cells infected with Mock or WT HCMV (MOI = 3), protein harvested at 48 hpi and processed by Western analysis (E). Cells pre-treated with TNFα (F) or IFNα (G) for 24 hr then infected with GFP-expressing wildtype HCMV (WT HCMV, MOI = 0.05). At 7 days post infection (dpi), viral spread was assessed by quantifying the total number of infected (GFP+) cells for each condition. Data normalized to control-treated ntg (100%). Data are represented as the mean of 6 biological replicates (A—D) or 12 biological replicates (F and G) +/-SEM as an XY-plot (left). Control pre-treatment is represented in the left segment of the plot as x = 0. Area under the curve was calculated for the right segment of each XY-plot, excluding the x = 0 control pre-treatment, and is represented as a bar graph (right). (A—D) p-values determined by student’s t-test n.s = not significant, *p<0.033, **p<0.002, ***p<0.001 (F and G) FDR-adjusted p-values determined using 2-way ANOVA followed by two-stage step-up method of Benjamini, Krieger and Yekutieli n.s = not significant, *p<0.033, **p<0.002, ***p<0.001.

Given PIAS1’s role in modulating cytokine signaling-associated gene expression, we examined how it contributes to cytokine-mediated inhibition of HCMV infection. PIAS1KO cells were significantly more sensitive to TNFα and IFNα with respect to their ability to limit WT HCMV infection relative to ntg controls (Fig 5A and 5B). In contrast, PIAS1 inactivation had little effect on IFNγ’s anti-viral activity (Fig 5C). The ΔUL26 virus is sensitive to TNFα pre-treatment [17], and this sensitivity is exacerbated by the inactivation of PIAS1 (Fig 5D), suggesting that in the face of TNFα treatment, the loss of PIAS1 compounds the cytokine-associated sensitivity of the ΔUL26 mutant. To target PIAS1 orthogonally, we utilized lentiviral-mediated CRISPRi to inhibit PIAS1 transcription [38,39] (Fig 5E). Cells expressing the more effective CRISPRi sg1 guide induced more significant anti-viral activity in response to TNFα and IFNα (Fig 5F–5G). Collectively, these experiments indicate that PIAS1 limits cytokine-induced anti-viral activities during HCMV infection.

### PIAS1 inactivation attenuates wildtype HCMV infection

**Fig 6:**
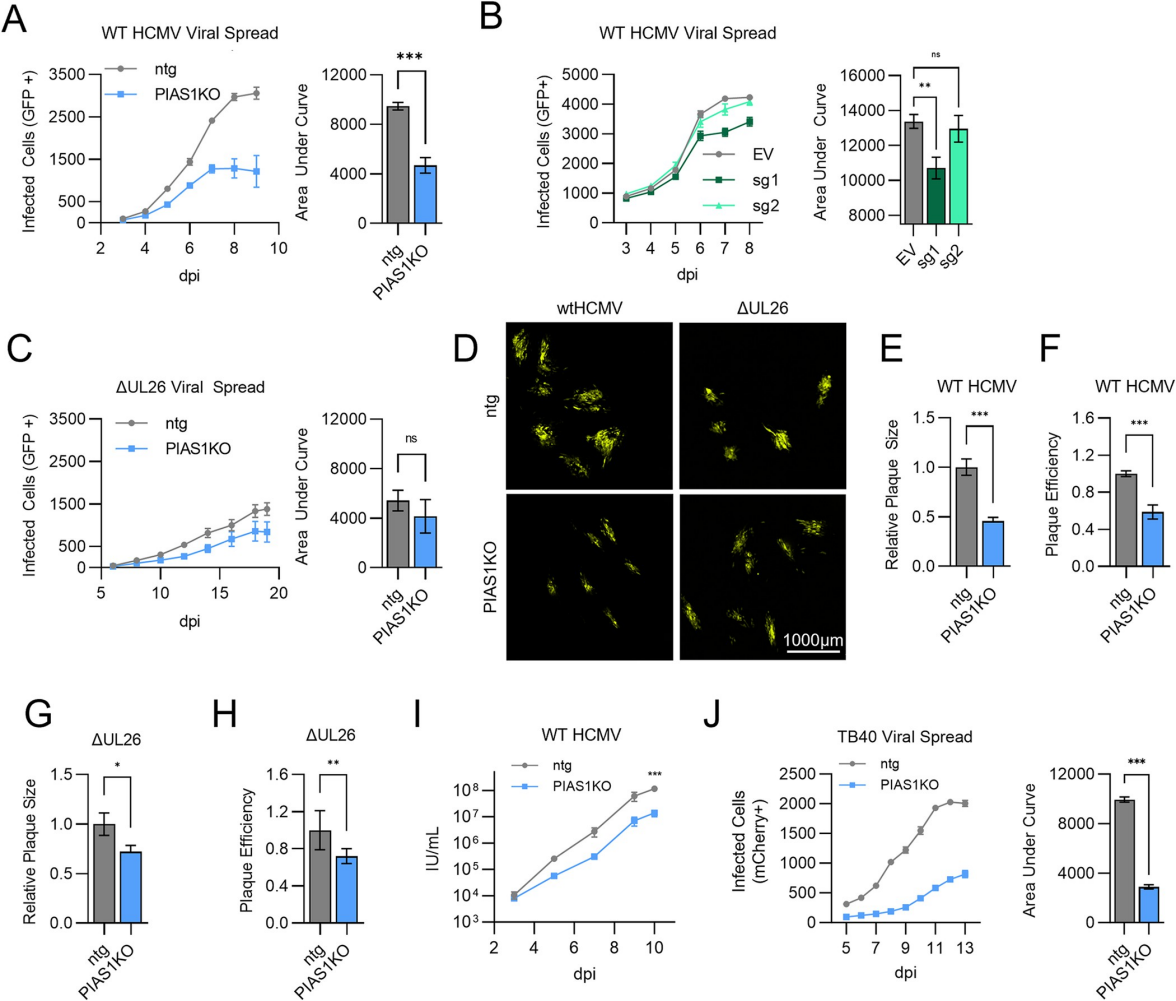
PIAS1 inactivation attenuates wildtype HCMV infection to a greater extent than ΔUL26 infection. (**A**) Viral spread of wildtype HCMV (WT HCMV) that expresses GFP at MOI = 0.01 in ntg (grey) and PIAS1KO (light blue) cells. Data represented as the mean of 6 biological replicates +/- SEM in an XY graph (left). Total area under curve (AUC) calculated for viral spread in each cell line and plot as a bar graph +/- SE (right). P-values determined student’s t-test n.s = not significant, *p<0.033, **p<0.002, ***p<0.001. (**B**) Viral spread of WT HCMV that expresses GFP at MOI = 0.05 in MRC5 cells transduced with CRISPRi plasmids containing empty vector (EV) or sgRNA targeting PIAS1 (sg1 dark green, sg2 light green). Data represented as the mean of 6 biological replicates +/- SEM in an XY graph (left). Total area under curve (AUC) calculated for viral spread in each cell line and plot as a bar graph +/- SE (right). FDR-adjusted p-values determined using 2-way ANOVA followed by two-stage step-up method of Benjamini, Krieger and Yekutieli n.s = not significant, *p<0.033, **p<0.002, ***p<0.001. (**C**) Viral spread of ΔUL26 that expresses GFP at MOI = 0.01 in ntg (grey) and PIAS1KO (light blue) cells. Data represented as the mean of 6 biological replicates +/- SEM in an XY graph (left). Total area under curve (AUC) calculated for viral spread in each cell line and plot as a bar graph +/- SE (right). P-values determined student’s t-test n.s = not significant, *p<0.033, **p<0.002, ***p<0.001. (**D**—**H**) Ntg and PIAS1KO cells infected with a low number of plaque forming units of WT HCMV or ΔUL26 that express GFP. (**D**) Representative images of plaques at 8 dpi. (E and G) Plaque size data are represented as the mean area of 20 representative plaques from each group normalized to the mean plaque size of wtHCMV in ntg cells +/- SE. (F and H) Plaque efficiency data are represented as the mean plaque count from 4 independent biological replicates +/- SE. (**E**—**H**) P-values determined student’s t-test n.s = not significant, *p<0.033, **p<0.002, ***p<0.001. (**I**) Viral replication of WT HCMV (GFP+) in ntg and PIAS1KO cells. Cells infected with WT HCMV (GFP+) (MOI = 0.1) and medium harvested from infections at the indicated times post infection (dpi) and then titered in MRC5 cells. Infectious units (IU) per mL was quantified for each sample and plotted as the mean +/- SEM of 4 biological replicates. P-values determined by student’s unpaired t-test, ns = not significant, *p<0.033, **p<0.002, ***p<0.001. (**J**) Viral spread of the HCMV clinical strain TB40 that expresses mCherry at MOI = 0.1 in ntg (grey) and PIAS1KO (light blue) cells. Infected cells (mCherry +) quantified by live-cell imaging every 24 hr starting at 5 days post infection (dpi) for 13 days. Data represented as the mean of 6 biological replicates +/- SEM in an XY graph (left). Total area under curve (AUC) calculated for viral spread in each cell line and plot as a bar graph +/- SE (right). P-values determined student’s t-test n.s = not significant, *p<0.033, **p<0.002, ***p<0.001.

Inactivation of PIAS1 reduced the number of infected cells in the absence of cytokine treatment (Fig 5A–5C). We next tested how PIAS1 contributes to HCMV viral spread. Inactivation of PIAS1 with either RNP or CRISPRi restricted WT HCMV spread (Fig 6A and 6B, respectively). In contrast, ΔUL26 viral spread was not significantly impacted by the absence of PIAS1 (Fig 6C), suggesting that the contribution of PIAS1 to infection depends on the presence of UL26. Plaque size and the initiation of WT HCMV infection were also both reduced in PIAS1KO cells compared to ntg cells (>50% reduction, Fig 6D–6F). PIAS1 inactivation also reduced the plaque size and initiation of infection for ΔUL26 but to a lesser extent (~25%, Fig 6D, 6G and 6H). Further, PIAS1 inactivation reduced the production of infectious virions during low MOI infection (Fig 6I), but did not appear to affect infection at high MOI (S7 Fig). We also examined the viral spread of TB40/e, a more clinically relevant strain of HCMV, and found that it was also restricted in PIAS1KO cells (Fig 6J). Collectively, our data indicate that PIAS1 is important for low MOI HCMV infection and that these contributions depend in part on the presence of UL26.

## Discussion

Innate immune evasion is an important determinant of successful viral replication. Viruses have developed strategies to evade detection and attenuate anti-viral signaling to support high titer replication. In this study, we find that the full-length UL26 protein proximally interacts with a complex composed of PIAS and STAT family members. Further, our data indicate that UL26 is necessary to broadly restrict STAT family member activation during infection and that UL26 is sufficient to attenuate cytokine-induced IFNα and TNFα signaling (Fig 2). Our results show that similar to UL26, the cellular PIAS1 protein is critical for limiting anti-viral gene expression during HCMV infection and upon cytokine challenge. Inactivation of PIAS1 substantially attenuates WT HCMV infection but has a reduced anti-viral impact on ΔUL26 infection, suggesting that UL26 is necessary for HCMV to benefit from PIAS1’s activity. Collectively, our data suggest that the UL26-PIAS1 complex attenuates anti-viral signaling during HCMV infection.

To identify UL26 interacting proteins that were more likely to be relevant to UL26’s contributions to HCMV infection, we compared the interactomes between TurboID-tagged full-length UL26 and a non-functional UL26 mutant, containing a 38-amino acid C-terminal deletion [19]. Of the 68 proteins preferentially biotinylated by full-length UL26, 67 were human proteins. Many of these were STAT and PIAS family members (Fig 1), which are well-known modulators of anti-viral signaling, and these interactions are consistent with UL26’s role in modulating cellular anti-viral defense. In addition, one viral protein was preferentially biotinylated by full-length UL26, UL31, which was ~8-fold enriched in the wildtype UL26 sample relative to the C-terminal deletion (S3 Table). UL31 can inhibit anti-viral signaling via cGAS inhibition [40]. This raises the possibility that UL26 and UL31 might cooperate to modulate anti-viral signaling during infection, although, for both UL26 and UL31, their expression in isolation is sufficient to attenuate anti-viral gene expression [17,40].

Previously, a global analysis of HCMV protein interactomes included the identification of proteins that interacted with stably expressed UL26 during infection [41]. The study identified 44 human proteins that putatively interact with UL26 [41], including two that we identified here, CREB-binding protein (CREBBP) and histone acetylase p300 (EP300). Both proteins have been found to interact with multiple partners in the STAT-PIAS complex depicted in Fig 1I. CREBBP and EP300 are intrinsically disordered proteins that modulate transcriptional activation, including the regulation of anti-viral gene expression [42,43]. While these two proteins were common between the earlier study and ours, the majority of the putative interactors were different in the two studies. This likely reflects the different timing of when the pulldown samples were processed. Here, we biotinylated proteins at twenty-four hours post-infection, a time at which UL26 localizes to the nucleus [15,19], whereas the previous interactome study performed pull-downs at sixty hours post-infection, a substantially later point of the viral life cycle, and a time at which UL26 is largely cytoplasmic [15,19,41]. These times reflect very different viral life cycle stages, and thus likely reflect differences in UL26 interactions, e.g., modulation of host gene expression in the nucleus versus viral packaging into the tegument that occurs in the cytoplasm. The previous interactome study also identified five UL26-interacting viral proteins [41], one of which, UL25, was also identified in our proximity-labeling experiment. UL25 was biotinylated by wildtype UL26 and UL26ΔC to a similar extent, suggesting this interaction is not dependent on the presence of the C-terminus of UL26. Little is known about the function of UL25 during infection. It is a nonessential protein located in the tegument [14,44], and is possibly involved in virion envelopment [45].

UL26 was found to interact with several PIAS and STAT proteins, and other innate immune modulators previously found to form complexes (Fig 1I) [24]. The known roles that these cellular proteins play in cellular immunity, coupled with UL26’s ability to block STAT and TNFα signaling strongly implicate these interactions as being important for HCMV-mediated inhibition of anti-viral defenses. We explored the UL26-PIAS1 interaction further and found that TurbioID-tagged PIAS1 could biotinylate UL26 in the absence of infection. This suggests that other viral proteins are not necessary for UL26 and PIAS1 to proximally interact. However, it remains to be elucidated which proteins UL26 is physically binding, and how UL26 binding might affect their activity. Despite these questions, UL26 and PIAS proteins can both broadly modulate both STAT and NFκB signaling [21,46], suggesting that a UL26-PIAS protein interaction may limit anti-viral signaling during infection.

The PIAS family of proteins are SUMO-ligases that contain a RING-finger-like zinc-binding domain (RLD) essential for their SUMO-ligase activity and a highly acidic (AD) domain that facilitates SUMO binding [20,47]. SUMOylation is a dynamic post-translational modification that regulates a number of molecular functions, including chromatin structure, DNA repair, transcription, innate immune signaling, cell cycle progression, protein trafficking, and the anti-viral response [27,46,48]. Here, we find that treatment with a SUMOylation inhibitor Subasumstat [32], attenuates HCMV replication (Fig 3E–3G). These data suggest that SUMOylation is broadly important for HCMV replication. However, our results suggest that PIAS1 inactivation did not impact global SUMOylation-induced by HCMV infection (S6 Fig), suggesting that HCMV infection induces the activation of other SUMO ligases to drive global increases in SUMOylation. UL26’s interaction with other PIAS family members (Fig 1) could be playing a role, but mammalian cells encode over two dozen SUMO ligases, so further interrogation of the responsible ligases is required. While pharmacological inhibition of SUMOylation attenuated HCMV replication, the pleiotropic effects associated with global inhibition of SUMOylation can make precise conclusions difficult as SUMO modifications have been found to have both pro- and anti-viral effects on HCMV infection [30]. The HCMV IE1 and IE2 proteins are SUMOylated during infection [49–53], as is the viral DNA polymerase processivity factor, UL44 [54,55]. In various instances, these SUMO modifications have been found to both support and inhibit infection, suggesting that the modulation of SUMOylation activity and the impact on infection is nuanced and context-specific.

Notably, PIAS1 can SUMOylate STAT1 at Lys703, which inhibits STAT1’s DNA binding and transcriptional activity following IFNγ-stimulation [33,56]. Similarly, PIAS proteins can inhibit NFκB signaling via binding and/or SUMOylating NFκB transcription factors and upstream regulators [35,57]. Further, SUMO-1 over-expression has been found to inhibit NFκB-dependent transcription [58]. We find that UL26 is sufficient to block IFNα and TNFα induced transcriptional activity (Fig 3), indicating that UL26 can broadly suppress anti-viral defenses. PIAS1 has exhibited similar activities, that is, inhibiting STAT and NFκB signaling [21,35,59]. Further, in over-expression experiments in the absence of infection, PIAS1 has been found to SUMOylate the HCMV IE2 protein, although the functional consequences during infection were not explored [53]. We found that the inactivation of PIAS1 on its own did not dramatically alter the expression of STAT or NFκB-responsive genes but instead modulated genes involved in cholesterol metabolism (Fig 4), a connection that has been previously observed [36,37]. However, upon stimulation by either HCMV infection or TNFα, cytokine signaling genes were strongly induced, partially phenocopying ΔUL26 infection (Fig 4), indicating that the lack of PIAS1 strongly potentiates anti-viral gene expression in the face of an inflammatory challenge. Consistent with this finding, the inactivation of PIAS1 made cells more resistant to viral infection, and more capable of limiting viral infection at lower doses of TNFα treatment (Fig 6). Notably, while the inactivation of PIAS1 substantially curtailed the viral spread of WT HCMV, there was no significant change in the spread of the ΔUL26 virus (Fig 6C). These results suggest that UL26 is necessary for HCMV to take advantage of the presence of PIAS1, consistent with a model in which a UL26-PIAS1 complex attenuates anti-viral gene expression.

Our results indicate that PIAS1 inactivation inhibits the viral spread of the HCMV TB40/e strain, a more clinically relevant variant relative to the heavily tissue culture-passaged AD169 strain. While several differences exist between the two strains, most notably, differences that impact entry and cellular tropism, the UL26 protein is almost identical, with a single difference at amino acid position 193, in which AD169 encodes a valine and TB40/e encodes an alanine. This likely suggests that UL26 functions similarly in both strains, although future experiments could examine this question, especially in different physiologically relevant cell types, e.g., epithelial, endothelial, and myeloid cells.

The specific mechanisms through which UL26 and PIAS1 modulate anti-viral gene expression remain to be elucidated. PIAS1 can alter transcription factor activity via transcription factor SUMOylation, direct binding and inhibition of transcription factors in the absence of SUMOylation, and through direct DNA binding at promoter sites, resulting in transcriptional repression [20,46]. It remains to be determined if UL26 specifically induces any of these mechanisms. Further, while we focused primarily on PIAS1, there are three additional human PIAS proteins, PIAS2, PIAS3, and PIAS4, that were all biotinylated preferentially by full-length UL26 and that have immunomodulatory activities [20,57,60]. It remains to be determined to what extent UL26 directly interacts with the various PIAS family members and modulates their activity.

Regulation of cellular inflammatory and anti-viral gene expression programs is a central host-pathogen interaction that governs host defense. Our results indicate that productive HCMV infection relies on UL26 and PIAS1 to prevent activation of anti-viral gene expression during infection. In this regard, HCMV has hijacked the host cell’s natural anti-inflammatory control mechanisms to limit anti-viral responses. A better understanding of the mechanisms involved could provide novel insights into these critical pathways and potentially highlight novel avenues for anti-viral therapeutic intervention.

## Materials and methods

### Tissue culture and virus propagation

Telomerase-transduced MRC5 lung fibroblasts and HEK293T cells were cultured in Dulbecco’s modified Eagle serum (DMEM; Invitrogen #11965118) supplemented with 10% (v/v) fetal bovine serum (FBS), and 1% penicillin-streptomycin (Pen-Strep; Life Technologies #15140–122) at 37°C in a 5% (v/v) CO_2_ atmosphere.

All AD169 viral stocks were derived from the BAd wt clone of AD169 Genebank accession number: FJ527563. The WT strain of HCMV used in experiments from BAd wt AD169 is referred to in this paper as WT HCMV. The GFP expressing wildtype AD169 is BADsubUL21.5. This virus is also referred to as WT HCMV with clarification in the figure legend that the GFP expressing strain was used [14]. The UL26-deletion virus is a GFP transposon insertion, herein referred to as ΔUL26 [14]. The truncated mutant of UL26 where the 185^th^ codon was replaced with a stop codon is herein referred to as UL26ΔC [19]. The TB40/E-mCherry virus was a generous gift from Christine M. O’Connor and Eain Murphy, generated as described here [61]. Recombinant viruses created for this work are described below in ‘BAC Recombineering’. All viral stocks used in this manuscript were propagated in MRC5 cells and concentrated by ultracentrifugation. Briefly, MRC5s were grown to confluence and placed in serum-free DMEM for 24 hr. Cells were infected at MOI = 0.01 (or 0.1 for viruses with a known growth defect, e.g. UL26-mutants). Infected cells were scraped into the medium and transferred to a sterile 50 mL conical. Cells were collected by centrifugation at 3,000 RPM for 5 minutes at 4C and virus-containing supernatant was set aside in a sterile Erlenmeyer flask. The cell pellet was resuspended in 5 mL virus-containing supernatant and transferred to a polystyrene tube. The cell solution was vortexed for 30 seconds and sonicated for 30 seconds three times then flash-frozen on dry ice and thawed in a 37C water bath to lyse cells. Cell debris was collected by centrifugation at 3,000 RPM for 5 minutes at 4C. The supernatant was combined with virus-containing supernatant in the Erlenmeyer flask. The medium was mixed well and distributed equally among ultracentrifuge tubes. Sorbitol buffer (20% D-sorbitol, 50 mM Tris-HCl, 1 mM MgCl_2_) was underlaid in each ultracentrifuge tube and the virus was collected at 26,000 rpm for 1.5 hrs at 23C. The supernatant was removed from the virus pellet by aspiration and the virus was suspended in a 1/25 volume ratio of original culture volume of 50:50 filter-sterilized solution of PBS supplemented with 3% BSA and DMEM supplemented with 10% FBS. Stocks were titered using a modified Reed & Muench TCID50 calculator from the Lindenbach lab [62].

For experiments involving infection, MRC5 cells were grown to confluence and then placed in serum-free DMEM supplemented with 1% PenStrep for 24 hours prior to infection. For experiments where cells are treated with cytokines or drugs prior to infection, MRC5 cells were grown to confluence and then placed in serum-free DMEM supplemented with 1% PenStrep and the indicated treatment for 24 hours prior to infection unless otherwise stated. To infect, the medium on cells was replaced with the minimum volume of viral adsorption medium to cover the monolayer of cells containing the indicated MOI in serum-free DMEM supplemented with 1% PenStrep. After 2.5 hr adsorption, the medium was removed and replaced with fresh serum-free DMEM supplemented with 1% PenStrep unless otherwise stated. Cells harvested as indicated at the timepoint of interest as described below.

### Reagents and preparation of treatments

Human tumor necrosis factor alpha (TNFα, GoldBio #1130-01-100), Interferon alpha (IFNα, Krackler # 45-I4276-20UG), and Interferon gamma (IFNγ, Medchemexpress #HY-P70610-50UG) were solubilized to 100 μg/mL concentration in sterile H2O and stored as 12 μL aliquots at -80C. Subasumstat (SUB, Fisher Scientific #50-217-3396) was solubilized to 10 mM in DMSO and stored at 20 μL aliquots at -80C. Biotin (Sigma, #B4501-100MG) was stored as a powder at room temperature and solubilized in serum-free DMEM to the indicated concentration immediately before use.

### Construction of plasmids

Primers used to assemble the fusion construct of UL26 to TurboID and clone into pLenti CMV/TO expression plasmid (addgene, #22262) are listed in S10 Table. The TurboID plasmid (3xHA-TurboID-NLS_pcDNA3, addgene #107171) and UL26 fragment were amplified to have homology arms to pLenti plasmid at the attb1/BamHI cut site and a 3x GGGGS linker repeat and purified. The pLenti vector was digested with BamHI (NEB #R0136) and XbaI (NEB #R0145) following the manufacturer’s instructions overnight. DNA fragments were combined with the digested pLenti plasmid in a Gibson Assembly reaction mix (NEB #M5510AA) at a molar ratio of 1:3:3 [vector:TurboID:UL26] for 1 hour at 50C. Following this reaction, STBL3 competent cells were transformed with 2 μL of the Gibson Assembly mixture and colonies were grown on LB agar plates supplemented with ampicillin at 37C overnight. Colonies were screened by colony PCR using primers that flank the construct insert region (See S10 Table, pLenti plasmid sequencing primers). DNA samples of the appropriate size were submitted to Azenta for Sanger Sequencing. Colonies containing plasmids with the appropriate sequence were grown in LB and stored in 50% glycerol at -80C.

### Bacterial artificial chromosome (BAC) recombineering

Recombinant virus containing TurboID fused to UL26 and UL26ΔC in the UL26 ORF were created as previously described [19] using the BAd wt clone of AD169 Genebank accession # FJ527563. The two-step PCR method for recombineering was followed as previously described using a BAd wt clone of AD169 with Kan/Isce I cassette in the UL26 ORF housed in GS1783 cells which contain an arabinose-inducible I-Sce 1 restriction site used for negative selection [19,63]. TurboID fused to UL26 wildtype or UL26ΔC were amplified to have homology arms to regions flanking the UL26 ORF of BAd WT clone of AD169, now containing Kan/Isce I cassette (BAd169+Kan), primers used are listed in S10 Table, BAC Recombineering Primers. Amplified DNA oligos containing TurboID fused to UL26 wildtype or UL26ΔC were then transformed into GS1783 electrocompetent cells containing BAd169+Kan and resulting colonies were negatively selected on LB +L-Arabinose plates in the presence or absence of kanamycin. Colonies that were no longer Kan-resistant were selected for colony PCR using a Touchdown method with primers flanking the UL26 ORF. Amplified DNA was run on an ethidium bromide agarose gel to identify colonies with DNA fragments of the expected size. These fragments were submitted to Azenta for Sanger Sequencing.

### Propagation of recombinant virus

Recombinant virus was isolated by growing GS1783 containing BAC of interest in 10 mL LB medium overnight. Cells were harvested by centrifugation at 4,000 RPM for 10 minutes at 4C. The cell pellet was resuspended gently in 200 μL cold buffer P1 from a Qiagen miniprep kit (#27106). Resuspension incubated at room temperature for 5 minutes. 200 μL buffer P2 from a Qiagen miniprep kit was added to the cell resuspension and incubated in ice for 5 minutes. Following this incubation period, 300 μL buffer P3 from a Qiagen maxiprep kit (#12163) was added to the sample tube and placed on ice for 10 minutes. The sample was centrifuged at 14,000 RPM for 10 minutes and supernatant was collected in a 2 mL sterile microcentrifuge tube. One mL of room temperature isopropanol was added to precipitate DNA and the tube was inverted 3x to mix. The sample was centrifuged at 14,000 rpm for 10 minutes at 4C. The supernatant was discarded and the pellet was resuspended in 500 μL filter-sterilized TEN buffer (25 mM Tris pH 7.6, 1 mM EDTA, 150 mM NaCl). The sample was incubated at room temperature for 10 minutes and then centrifuged at 14,000 rpm for 30 seconds. The supernatant was transferred to a clean 1.5 mL microcentrifuge tube and 1 mL prechilled 100% ethanol was added to the supernatant. The sample was inverted 3x and centrifuged at 14,000 RPM for 10 minutes at 4C. The supernatant was discarded and open tubes were placed in a sterile hood for 5 minutes for ethanol to evaporate. DNA was suspended in 50 μL sterile double-deionized water.

MRC5s were transfected with isolated BAC DNA. At 70–80% confluence, MRC5 fibroblasts were trypsinized and cells were isolated by centrifugation at 1.5 rpm for 5 minutes at 4C. The supernatant was discarded and cells were resuspended in 500 μL solution 6 (140 mM Na phosphate buffer, 5 mM KCl, 10 mM MgCl2, pH ~7.2). 25 μL BAC DNA prep and 1 ug pp71 expression plasmid were added to the cell suspension. The sample was transferred to a 4 mm electroporation cuvette and cells were electroporated using BioRad Gene Pulser Xcell total system (#1652266) set to exponential, 265 V, 960 μF to yield a time constant between 15 and 20. Immediately after transfection, the electroporation mixture was added to a sterile 10 cm tissue culture dish containing 8 mL DMEM supplemented with 10% FBS and 10 mM Na Butyrate. Cells were placed in a 37C incubator for 2 hours to settle, then the medium was aspirated and replaced with fresh DMEM supplemented with 10% FBS. Cells were placed in a 37C incubator overnight and monitored every day until confluent. Once confluent, cells were transferred to a 15 cm tissue culture dish by trypsinization and grown to confluence. Cells were monitored every day for signs of infection and the virus was harvested from cells 2 days after 100% cytopathic effect (CPE) was apparent as described above.

### Proximity proteomics sample preparation and analysis

For each condition, approximately 1e7 MRC5 cells were grown to confluence and placed in serum-free DMEM medium for 24 hours. Cells were mock infected or infected with TurboID-N-UL26wt or TurboID-N-UL26ΔC (MOI = 3) using 9 mL of viral adsorption medium for 2.5 hours. The adsorption medium was removed and replaced with serum-free DMEM. At 23 hours post-infection, the medium was replaced with serum-free DMEM supplemented with 1 μM Biotin. After 1 hour of incubation with biotin, the medium was removed and cells were washed 2x with cold PBS. Cells were harvested in 5 mL RIPA lysis buffer (50 mM Tris pH 7.4, 150 uM NaCl, 1 mM EDTA, 0.25% (wt/v) Sodium Deoxycholate, 1% (v/v) TritonX-100, and 0.1% SDS) and transferred into a chilled 15 mL centrifuge tube, vortexed for 30 seconds and placed on ice for 20 minutes. Lysates were vortexed for 30 seconds and centrifuged for 15 minutes at 14,000 rpm at 4C.

To prepare beads for affinity pulldown, 100 μL streptavidin-bound magnetic beads (Dynabeads MyOne Streptavidin T1, ThermoFisher #A1254302) per sample were washed with 5x volume RIPA without detergent 3 times for 5 minutes each wash. Beads were resuspended in the original volume and kept on ice until ready to use.

Samples were incubated with 100 μL prepared magnetic beads for 1 hour at room temperature. The supernatant was removed from each sample and beads were washed with 1 mL Wash Buffer 1 (2% SDS in H2O) for 5 minutes on a rotator. The supernatant was removed and beads were washed with 1 mL Wash Buffer 2 (50 mM HEPES pH 7.5, 500 mM NaCl, 1 mM EDTA, 0.1% (v/v) TritonX-100) for 5 minutes on a rotator. The supernatant was removed, and beads were washed with 1 mL Wash Buffer 3 (10 mM Tris-HCl pH 7.4, 250 mM LiCl, 1 mM EDTA, 0.1% (wt/v) sodium deoxycholate) for 5 minutes on a rotator. The supernatant was removed and beads were washed with 1 mL 50 mM Tris (pH 7.4) for 5 minutes on a rotator. The supernatant was removed and protein was eluted from the beads with 40 μL Disruption Buffer (2% SDS, 50 mM Tris pH 7, 5% β-mercaptoethanol, 2.5% (v/v) Glycerol, Bromophenol Blue). Samples were boiled in Disruption Buffer for 5 minutes and centrifuged at 14,000 RPM for 10 minutes. The supernatant was transferred into a new Eppendorf tube, 10 μL affinity pulldown sample was run on a 10% acrylamide SDS-PAGE gel at 115 V for 1.5 hours. This gel was stained with Pierce silver stain for mass spectrometry (#B4501). The remaining 30 μL sample was run on a 10% acrylamide SDS-PAGE gel at 100 V for 15 minutes and stained with Coomassie (Bio-Rad #P1610786) following the manufacturer’s instructions. The gel was brought to the University of Rochester Proteomics core for sample prep and analysis.

Gel bands were excised, cut into 1 mm cubes, de-stained, then rinsed and alkylated with DTT and IAA. Gel pieces were dehydrated with acetonitrile then covered with a minimum volume of trypsin which had been reconstituted to 10 ng/uL in 50 mM ammonium bicarbonate for 30 minutes at room temperature. Samples were then submerged completely in trypsin solution overnight at 37°C. Peptides were extracted by the addition of 50% acetonitrile and 0.1% TFA, then dried down in a CentriVap concentrator (Labconco). Peptides were desalted with homemade C18 spin columns, dried again, and reconstituted in 0.1% TFA.

Peptides were injected onto a homemade 30 cm C18 column with 1.8 um beads (Sepax), with an Easy nLC-1200 HPLC (Thermo Fisher), connected to a Fusion Lumos Tribrid mass spectrometer (Thermo Fisher). Solvent A (0.1% formic acid in water) solvent B (0.1% formic acid in 80% acetonitrile). Ions were introduced to the mass spectrometer using a Nanospray Flex source operating at 2 kV. The gradient began at 3% solvent B and held for 2 minutes, increased to 10% solvent B over 5 minutes, increased to 38% solvent B over 38 minutes, then ramped up to 90% solvent B in 3 minutes and was held for minutes, before returning to starting conditions in 2 minutes and re-equilibrating for 7 minutes, for a total run time of 60 minutes. The Fusion Lumos was operated in data-dependent mode, with MS1 scans acquired in the Orbitrap, and MS2 scans acquired in the ion trap (cycle time 1.5 seconds). Monoisotopic Precursor Selection (MIPS) was set to Peptide. The full scan was done over a range of 375–1400 m/z, with a resolution of 120,000 at m/z of 200, an AGC target of 4e5, and a maximum injection time of 50 ms. Peptides with a charge state between 2–5 were picked for fragmentation. Precursor ions were fragmented by collision-induced dissociation (CID) using a collision energy of 30% with an isolation width of 1.1 m/z. The Ion Trap Scan Rate was set to Rapid, with a maximum injection time of 200 ms, an AGC target of 1e4. Dynamic exclusion was set to 20 seconds.

SEQUEST search engine was used to search raw data within the Proteome Discoverer software platform, version 2.4 (Thermo Fisher), using the SwissProt human and HHV-5 databases. Trypsin was selected as the enzyme allowing up to 2 missed cleavages, with an MS1 mass tolerance of 10 ppm, and an MS2 mass tolerance of 0.6 Da. Carbamidomethyl was set as a fixed modification, while oxidation of methionine was set as a variable modification. The Minora node was used to determine relative protein abundance between samples using the default settings. Percolator was used as the FDR calculator, filtering out peptides which had a q-value greater than 0.01.

The sum of the peak areas for each peptide that could be integrated for each protein were represented as abundance for each protein. Samples with non-detected abundance values were replaced with the minimum abundance detected for the data set (1.03e4, 912 values replaced). Each abundance value was normalized to the median abundance value for that sample and multiplied by 1e7. Normalized values for Tur-N-UL26WT +Biotin and Tur-N-UL26ΔC +Biotin were graphed together as a scatter plot (Fig 1H) with a line of identity (m = 1).

Human proteins 5-fold enriched in Tur-N-UL26WT+Biotin relative to Tur-N-UL26ΔC (67 proteins) were uploaded to the Human Reference Protein Interactome Mapping Project (query-query) [24] to identify an interactome of 12 proteins that were preferentially biotinylated by Tur-N-UL26WT. These data were used to create Fig 1I. The list of Human proteins 5-fold enriched in Tur-N-UL26WT+Biotin relative to Tur-N-UL26ΔC (67 proteins) was also analyzed by reactome.org Analysis Tool [64]. The gene list was projected to human identifiers to provide the data in S4 Table. Entities FDR values were -log_10_ transformed to provide the data in Fig 3A.

### Transfection of 293T cells

293T cells were grown to 60% confluence in a 10 cm dish. Plasmid transfection mix was prepared by bringing all reagents to room temperature. A total of 57 μg plasmid was combined with 45.6 μL PEI reagent in serum-free DMEM. Plasmid transfection mix was incubated at room temperature for 20 minutes and then added dropwise to the appropriate 10 cm dish. Cells incubated with the plasmid transfection mix overnight. Transfected cells were trypsinized and split equally between three 10 cm dishes. Cells settled overnight. At 48 hours post transfection, cells were treated with 1 μM Biotin for 1 hour and processed as indicated above for streptavidin affinity purification. Prior to bead incubation, these lysates were treated with Benzonase and 1 mM MgCl_2_ then incubated with rotation at 4°C for 4 hours.

### CRISPR Ribonucleoprotein (RNP)-based knockout

Gene disruption by CRISPR-Cas9 ribonucleoprotein (RNP) was performed with the Neon Transfection System 10 μL kit (ThermoFisher #MPK1025) as described previously [65] with electroporation settings voltage: 1100 V, width: 30 ms, pulses: 1. Guide RNAs were ordered from Synthego:

Negative Control Scrambled sgRNA (modified) #1: 5’-GCACUACCAGAGCUAACUCA-3’

RELA (sgRNA EZ kit): 5’-UCCCCUGGCAGAGCCAGCCC-3’

STAT1 (Gene Knockout Kit v2): sgRNA1: 5’-AGUGGUUAGAAAAGCAAGAC-3’; sgRNA2: 5’-AAAGCUGGUGAACCUGCUCC-3’; sgRNA3: 5’-UUCCCUAUAGGAUGUCUCAG-3’

PIAS1 (Gene Knockout Kit v2): sgRNA1: 5’-GCCAGCCUUUAGCAAAUGCA-3’; sgRNA2: 5’-CAGGCGUCAUGAUUUUCUGU-3’; sgRNA3: 5’-GGAGACAAAGUUGCUGGCAU-3’

### CRIPSRi gene knockdown

Two independent guide RNAs directed to the PIAS1 gene locus (sg 5’-CGGACAGTGCGGAACTAA-3’, sg2 5’-TAGGGAGTCCGGAGGTAG-3’) were designed with a 5’ and 3’ flanking sequence to a CRISPRi Lentivirus (LV) PURO plasmid (pLV hU6-sgRNA hUbC-dCas9-KRAB-T2a-puro with stuffer), gifted by the Harris Lab at University of Rochester Department of Biomedical Genetics (5’-GGAAAGGACGAAACACCG-3’ & 5’-GTTTTAGAGCTAGAAATAGC-3’, respectively). Guides with flanking sequences to the CRISPRi LV plasmid were amplified by PCR, cloned into the CRISPRi plasmid by Gibson Assembly as described above, and transformed into STBL3. Plasmids isolated from positive colonies were screened by Sanger Sequencing to verify the PIAS1 sgRNA sequence. Lentivirus was propagated in HEK293T cells and used to transduce MRC5s as previously described [16].

### Western blotting

Protein detection was performed as previously described [66]. The following antibodies were used for western blot analysis following the manufacturer’s instructions: STAT1 (Cell signaling, #D1K9Y), Phospho-STAT1 (Cell Signaling, #58D6), STAT2 (Cell Signaling, #D9J7L), Phospho-STAT2 (Cell Signaling, #D3P2P), GAPDH (Cell Signaling #5174), STAT3 (Cell Signaling #79D7), Phospho-STAT3(Tyr705) (Cell Signaling #D3A7), HA-Tag (Cell Signaling #2367), PIAS1 (Cell Signaling #D33A7), PIAS2 (ABclonal, #A5654), PIAS3 (Cell Signaling, #9042), PIAS4 (Cell Signaling, #4392), SUMO1 (Cell Signaling, #4930S), SUMO2/3 (Cell Signaling, #4971S), viral proteins IE1 [67], pp28 [68], UL26 [15] (Mouse mAb).

For the detection of biotinylated proteins, cell lysates were run on a gradient SDS-PAGE gel and transferred onto a nitrocellulose membrane as described above. The membrane was then blocked in 1X TBST supplemented with 1% BSA and 0.2% TritonX100 for 10 minutes. The membrane was incubated in a diluted streptavidin conjugated to HRP (Fisher, PI-21130) solution (1:40,000 in 1xTBST supplemented with 1% BSA) for 40 minutes. Following this incubation, the membrane was washed 3x with 15 minutes with 1xTBST and imaged as described above.

For quantification of western blots, band intensities for GAPDH and protein of interest were determined using Image Lab v4.1 for each lysate. GAPDH intensity for all samples was divided by the maximum GAPDH intensity. Band intensity of the protein of interest was then normalized to the calculated GAPDH value and expressed as a percentage.

### Luciferase assay

The ISRE firefly luciferase reporter plasmid was a gift from the lab of Dr. Ruth Serra-Moreno. HEK293T cells were seeded in white-walled 96 well plates. At 30% confluence, cells were treated with 1 μg total DNA (250 ng firefly expression plasmid, 250 ng renilla expression plasmid, 500 ng EV or UL26) and 1.2 μL Fugene transfection reagent. At 48 hr, treatments (IFNα or TNFα) were added to the appropriate samples. 24 hours later, luciferase assays were carried out as previously described [17] using a Promega dual luciferase reporter assay.

### Virion production by TCID50

The TCID50 method was used for characterizing the replication of viral strains that do not express GFP (e.g. UL26ΔC and the recombinant viruses containing TurboID). Cells were treated and infected as described above with the indicated virus. At the desired time post-infection, the medium was collected from the cell monolayer, frozen on dry ice in cryovials, and stored at -80C. Samples were thawed in a 37C water bath, vortexed, and then centrifuged for 5 minutes at 3,000 RPM to pellet cellular debris. Virus-containing supernatants were serially diluted (1:10) eight times in a deep 96-well plate. Using a multichannel pipette, 50 μL of each dilution was transferred to each well within a row of a 96-well plate containing sub-confluent MRC5 cells in 50 μL growth medium (n = 12). After 2–3 days post-infection, 100 μL serum-free DMEM was added to each well. Infected wells were identified 10 days post-infection and sample titers were calculated using a modified Reed & Muench TCID50 calculator from the Lindenbach lab [62].

### Plaque efficiency and size assay

Plaque assays were performed in the indicated cell type as previously described [65]. At 8–10 dpi, plaques were imaged using a Cytation 5 imaging reader (BioTek). Field of view (FOVs) with plaques were manually captured using a 4x magnification objective lens and predefined YFP channel with an excitation wavelength of 469 nm and emission wavelength of 525 nm for GFP+ cells. For each condition, at least 20 independent plaques were captured. Plaque-size objects within each FOV were defined with Gen 5 Image Primer 3.11 software on the YFP image as objects at least 100 μm in size and intensity threshold of 5,000 units, touching objects were not split. These experiments used GFP-expressing WT HCMV and ΔUL26, rather than the UL26ΔC mutant which does not express GFP, to improve the accuracy of the assay.

### Viral spread

This assay reports viral spread by quantifying GFP+ cells from a low MOI infection over multiple days in an infected TC well. MRC5s were grown to confluence in 96-well TC-treated black-walled plates and treated/infected as indicated. To detect infected (GFP+) cells over time, plates were imaged using Cytation 5 imaging reader (BioTek) using a 4x magnification objective lens and predefined YFP channel with an excitation wavelength of 469 nm and emission wavelength of 525 nm. Infected (GFP+) cells were defined with Image Primer 3.11 software on the YFP image as objects 20–200 um in size with an intensity threshold of 5,000 units, touching objects were split. For viral spread assays, GFP+ cells were quantified every 24 hours and data was represented as infected cell (GFP+) counts for each day. For cytokine pre-treatment assays, GFP+ cells were quantified at 6 dpi for WT HCMV and 7 dpi for UL26TI. All samples were normalized to the number of infected (GFP+) cells in the untreated ntg control condition and expressed as a percent of 100.

### Fluorescent viral replication quantification (Infectious Units/mL)

MRC5 cells were seeded into a 384-well black-walled plate (45 uL) and grown to confluence. Virus samples from WT-GFP or ΔUL26 infections were loaded into rows A-D of a 96-well plate. Using an Integra Assist Plus instrument and 12-channel integra electric pipette, 5 μL of each virus sample from the 96 well plate was transferred to the 384 well plate and serially diluted 1:10 four times.

At 48 hpi, the number of infected cells in each well was determined using Cytation 5 imaging reader (BioTek) using a 4x magnification objective lens and predefined YFP channel with an excitation wavelength of 469 nm and emission wavelength of 525 nm. Infected (GFP+) cells were defined with Image Primer 3.11 software on the YFP image as objects 20–200 um in size with an intensity threshold of 5,000 units, touching objects were split. The total number of infected cells was considered ‘infectious units’ and the total infectious units/mL for each sample was calculated from the appropriate dilution.

### Viral DNA accumulation

Cells were grown to confluence in a 12 well TC plate and infected as described above. Cells were harvested at the indicated time post infection in 150 μL Lucigen QuickExtract DNA Extraction Solution. DNA was extracted following the manufacturer’s protocol. DNA was diluted 1:1000 in sterile H2O and analyzed by RT-qPCR. Relative quantities of IE1 compared to GAPDH were calculated as previously described [70] and normalized to the mean of ntg_24hpi vDNA abundance [1].

### Click-Seq library construction and data analysis

First, 5 μg total RNA was subjected to poly(A) mRNA isolation according to the manufacturer’s protocols (NEB, #E7490). Then, 10 μL of the eluted mRNA was mixed with 1 μL 100 μM Genomic Adaptor_6N primer (5’-GTGACTGGAGTTCAGACGTGTGCTCTTCCGATCTNNNNNN-3’) and 2 μL of 5 mM AzNTP (Baseclick, #BCT-25~ -28)/dNTP mixture in a 1:35 ratio. The mixture was then heated at 65°C/5 min and snap-cooled on ice for 3 min. Then, the reverse transcription was carried out using SuperScript III (ThermoFisher, #18080093). In brief, 7 μL master mix containing 4 μL 5X Superscript First Strand Buffer, 1 μL 0.1 M DTT, 1 μL RNaseOUT (ThermoFisher, #10777019), and 1 μL Superscript III was mixed with the RNA sample. Heating was performed as follows: 25°C/10 min, 50°C/40 min, and 75°C/15 min sequentially in thermocycler followed by RNase H (ThermoFisher, #AM2293) treatment using 1 U per reaction for 37°C/30 min and then 80°C/10 min. The cDNA was then purified using Sera-Mag Speedbeads (Cytiva, #65152105050250). Speedbeads working solution was made by washing 1 mL beads slurry twice in 1XTE buffer and then resuspending in 50 mL of 1XTE buffer containing 9 g PEG-8000, 1 M NaCl, and 0.05% Tween-20. Speedbeads, 1.8x reaction volume, was mixed with cDNA followed by a 5 min incubation at room temperature. The beads were then pelleted using a magnetic bead collector and the supernatant was discarded. Two washes with 80% ethanol were performed while the beads were pelleted on the magnet. The beads were then dried, and the cDNA was eluted by resuspending the beads in 22 μL 50 mM HEPES pH 7.2 for 2 min at room temperature. Then, 20 μL of the cDNA was mixed with 11 μL Click Mix and 4 μL of 5 μM UMI-click-adapter (5’-Hexynyl-NNNNNNNNNNNNAGATCGGAAGAGCGTCGTGTAGGGAAAGAGTGT-3’, HPLC purified). To activate the reaction, 4 μL of 50 mM vitamin C and 1 μL of Click Catalyst were pre-mixed before being added into the cDNA solution for 60min room temperature incubation in the dark. Clicked cDNA was then purified in the same manner as post-RT purification except eluted in 22 μL of 10 mM Tris pH 7.4. Then, half of eluted cDNA was subjected to PCR amplification with indexing primers i5 (5’-AATGATACGGCGACCACCGAGATCTACAC[Index]ACACTCTTTCCCTACACGACGCTCTTCCGATC*T-3’) and i7 (5’-CAAGCAGAAGACGGCATACGAGAT[Index]GTGACTGGAGTTCAGACGTGTGCTCTTCCGAT*C-3’). Both primers have a phosphorothioate bond added at the 3’ end (denoted as *) to increase stability. The PCR reaction was carried out in the volume of 50 μL including 25 μL 2X OneTaq Master Mix (NEB, #M0482), 2 μL of 5 μM i5 and i7 each, and cDNA in H_2_O with a total of 15 cycles of amplification given the 5 μg input RNA. The program was run at 94°C/4 min; 53°C/30 sec; 68°C/10 min; 15X [94°C/30 sec, 53°C/30 sec; 68°C/2 min]; 68°C/5 min; hold at 4°C. The library was then purified and size-selected with Speedbeads. In brief, 45 μL beads (0.9X volume of PCR reaction) were mixed with the reaction to remove larger fragments. The supernatant was collected and mixed with 10 μL beads (0.2X volume of PCR reaction) to remove smaller fragments and primer dimers. Two washes with 100% ethanol were then performed while the beads were on the magnet. Beads were dried before being resuspended in 12 μL of 10 mM Tris pH 7.4. The library was then collected and subjected to quality control on Agilent 2200 TapeStation and to analysis on Illumina NovaSeq 6000 at the Genomics Research Core at the University of Rochester. Data was reported using Multi v 1.11 [69]. Data was formatted with bcltofastq-2.19.1 and cleaned with fastp 0.23.1. Sequence reads were mapped to the human reference genome (GRCh38 + Gencode-38 annotation) using STAR_2.7.9a.

Protein coding transcripts were isolated from the data for further analysis (S5 and S6 Tables). For genes with multiple ensemble transcript accession numbers, the sample with the largest base mean value was selected to represent that gene. Genes with Log2 Fold Change > 2 and padj < 0.5 were analyzed by Reactome analysis tool as described above [64] to provide the data in S6 and S7 Tables and Fig 4C–4G, and was submitted to the Gene Expression Omnibus (GEO), GSE264589. Previously reported data (GSE137065) were re-analyzed using the same methods (see S8 Table and Fig 4E, 4F, and 4H). Data from this work (S7 Table, GSE264589) were compared to previously reported data (GSE137065, S8 Table) in S9 Table. Intersecting genes were identified to produce the data represented in Fig 4E and 4F.

### Analysis of RNA (RT-qPCR)

RNA was isolated from cells by Trizol extraction and used to synthesize cDNA with a qScript cDNA synthesis kit (Quantabio). Samples were analyzed by Real-Time qPCR and relative quantities of gene expression were calculated as previously described [70]. The following primers were used:

BST2 F 5’-AACACGGTTAGCGGGGAGAG-3’; BST2 R 5’-GGGACTCATTGTCCGGAGGG-3’; ISG15 F 5’-CGGGCAACGAATTCCAGGTG-3’; ISG15 R 5’-CACGCCGATCTTCTGGGTGA-3’; IFIT3; F 5’-GGAAACAGCCATCATGAGTGAGG-3’; IFIT3 R 5’-TGAATAAGTTCCAGGTGAAATGGC-3’; GAPDH F 5’-CATGTTCGTCATGGGTGTGAACCA-3’; GAPDH R 5’-ATGGCATGGACTGTGGTCATGAGT-3’

### Immunofluorescence staining, imaging, and analysis

Cells were grown to confluence on 0.2 x 0.2 mm glass coverslips in 6 well TC dishes and treated as described. At the indicated time post infection, cells were fixed in PBS supplemented with 2% paraformaldehyde for 15 minutes in the 6 well TC dish. Coverslips were washed 3x with PBS and permeabilized in PBS supplemented with 0.1% tritonX-100 and 0.1% SDS. Cover slips were washed 3x with PBS and cells were blocked with PBS supplemented with 2% BSA, 0.05% Tween-20, 0.3% TritonX-100, and 5% Human Serum for 1 hour at room temperature. Coverslips were transferred onto slips of parafilm containing a 50 μL drop of the indicated antibody (α-HA, cell signaling #C29F4, 1:800) diluted in PBS supplemented with 0.01% Tween-20 for 1 hour at room temperature. Cover slips were transferred to a clean 6 well TC dish for 3x 15 minute washes in PBS supplemented with 0.01% Tween-20. Cover slips were transferred onto slips of parafilm containing a 50 uL drop of Alexa flour 594 goat-anti-rabbit IgG (1:1000) prepared in PBS supplemented with 0.5% Tween-20 for 1 hour. Cover slips were transferred to a clean 6 well TC dish for 3x 15 minute washes in PBS supplemented with 0.01% Tween-20. The final wash was supplemented with Hoechst 33342 (Invitrogen #H3570). Coverslips were mounted onto glass slides containing a 50 μL drop of 90% glycerol. Cover slips were secured with clear nail polish and left to dry in the dark.

Coverslips were imaged using Cytation 5 imaging reader (BioTek) using a 20x magnification lens. Fluorescence was measured with a predefined Texas Red channel with an excitation wavelength of 586 nm and emission of 647 nm, and a predefined DAPI channel with an excitation wavelength of 377 nm and emission of 447 nm.

### Cell proliferation

Approximately 2,000 cells were seeded in 96 well plates and allowed to settle overnight at 37°C. At 24, 48, and 72 hpi; nuclei were stained with Hoechst in n = 12 wells and quantified using Cytation 5 imaging reader (BioTek) using a 4x magnification lens. Fluorescence was measured with a predefined DAPI channel with an excitation wavelength of 377 nm and emission of 447 nm. Exponential (Malthusian) growth curve analysis from GraphPad Prism v10.0.1 was applied to the data to produce doubling time values.

### Statistics

All statistics were carried out using GraphPad Prism v10.0.1 unless otherwise indicated.

## Supporting information

S1 Fig Dynamic STAT phosphorylation in early HCMV infection. MRC5 cells infected with WT HCMV or UL26ΔC (MOI = 3). Protein harvested at the indicated hour post infection (hpi) and processed by western blot as indicated.
(TIF)

S2 Fig Subasumstat treatment does not impact nuclei count over multiple days of treatment. Nuclei count of MRC5 cells infected with Mock (grey) or HCMV that expressed GFP (MOI = 0.05, red) and treated with a 10-point dose curve of Subasumstat at 6 days post treatment (n = 6). Nuclei count of control wells (0 uM, Mock or HCMV) are represented as horizontal dashed lines.
(TIF)

S3 Fig UL26 and TurboID-PIAS1 expression and biotinylation optimization in 293T cells. (A-B) HEK293T cells transfected with HA-TurboID-PIAS1 (HA-Tur-P1), HA-TurboID-NLS (HA-Tur-NLS), UL26 WT, UL26ΔC, or empty vector (EV) as indicated. At 47 hours post transfection, cells were treated with the indicated concentration of Biotin. Protein harvested from cells at 48 hours post transfection (1 hr biotin incubation) and processed by western blotting. (A) Expression of TurboID and UL26 proteins. (B) Total protein biotinylation (top) Ponceau stain (bottom).
(TIF)

S4 Fig Extraction of biotinylated proteins from lysates by streptavidin affinity purification. HEK293T cells were transfected with HA-TurboID-PIAS1 (HA-Tur-PIAS1), HA-TurboID-NLS (HA-Tur-NLS), UL26 WT, or UL26ΔC as indicated. At 24 hours post transfection, cells were treated with vehicle or 50 ng/mL TNFα. At 47 hours post transfection, cells were treated with Biotin (1 μM). Protein harvested from cells at 48 hours post transfection (1 hr biotin incubation) and lysed using RIPA buffer for streptavidin-based affinity purification as indicated in materials & methods. Input samples (In) were collected immediately prior to affinity purification. Unbound samples (U) were collected immediately following incubation with streptavidin-bound magnetic beads. Proteins in input and unbound samples were separated by SDS-PAGE and transferred onto nitrocellulose membranes. Membranes were stained with Ponceau to visualize total protein (bottom), destained with TBST, and processed by western blotting for avidin-peroxidase (top).
(TIF)

S5 Fig Ntg and PIAS1KO Cellular proliferation. Proliferation rates of MRC5 cells treated with CRISPR RNPs containing non-targeting guide (ntg) or PIAS1 sgRNAs (PIAS1KO). Doubling time (hours) calculated as indicated in materials & methods.
(TIF)

S6 Fig Impact of PIAS1 inactivation on SUMOylation. Ntg and PIAS1KO cells infected with Mock, WT HCMV (WT), or UL26ΔC (MOI = 3, n = 2). Protein harvested at 48 hpi and processed by Western analysis as indicated.
(TIF)

S7 Fig Impact of PIAS1 inactivation on high MOI infection. (A-C) Ntg and PIAS1KO MRC5 cells infected with WT HCMV (MOI = 3). Protein (A, n = 1), mRNA (B, n = 3), viral DNA (C, n = 3) was collected and processed for Western analysis (A) or RT-qPCR (B & C). (D) Ntg and PIAS1KO MRC5 cells infected with GFP-expressing WT HCMV (MOI = 3). Virus-containing media samples were harvested at the indicated times post infection and titered in MRC5 cells, titers are represented as IU/mL (D). (B & C) FDR-adjusted p-values determined using 2-way ANOVA followed by two-stage step-up method of Benjamini, Krieger and Yekutieli n.s = not significant, *p<0.033, **p<0.002, ***p<0.001.
(TIF)

S1 Table Proximity proteomics data. Proteomics data from biotin-enriched samples harvested as outlined in Fig 1F. Description of columns: Protein FDR Confidence: The confidence that the software has that the given protein is present in the sample. In most cases, we filter the data to show only high confident proteins. Accession: Uniprot or NCBI accession number. Description: Name of the protein, along with the species and gene name. The gene name can be found after “GN =“. Marked As: This column is present if multiple species were searched. It will show which database the given protein originated from. MW [kDa]: Molecular weight of the protein. # of Unique Peptides: Peptides can be shared between different proteins or protein isoforms, so this column shows the number of peptides that are unique to the given protein group. # of Peptides: The total number of unique and shared peptides for the given protein group. # of PSMs (Peptide Spectral Matches): Peptide spectral matches are MS2 scans that are matched to a peptide by the search algorithm. This differs from the number of peptides because the same peptide can be fragmented and identified multiple times within a mass spec run, especially if the protein that the peptide originated from was in high abundance. The number of PSMs will therefore always be greater than or equal to the number of peptides identified. Abundance Ratio (log2): The protein fold change in log2 format between the given samples or groups. The software caps the maximum fold change at 100, so the largest log2 fold change you will see is 6.64 (2^6.64 = 100). Abundance Counts: The number of peptides that are used to calculate the abundance of the given protein. Abundances (or Normalized Abundances): The sum of the peak areas for each peptide that could be integrated for the given protein. If normalization was selected, then the normalized abundances will be shown.
(XLSX)

S2 Table Normalized proximity proteomics data. Median normalized protein abundance, from S1 Table, supplied data for Fig 1H. Proteins 5-fold enriched in WT+B relative to UL26ΔC+B (dark green, right).
(XLSX)

S3 Table HCMV proteins detected in proximity proteomics experiment. HCMV proteins from S2 Table. Proteins 5-fold enriched in WT+B relative to UL26ΔC+B (dark green, right).
(XLSX)

S4 Table Reactome analysis of genes enriched in WT UL26 sample. Reactome analysis on the list of proteins 5-fold enriched in WT UL26 compared to UL26ΔC from proximity proteomics analysis, input list is from S2 Table represented in green (left). Reactome analysis report (right). The top 10 pathways scored by -log_10_(Entities FDR) are highlighted in orange and are represented in Fig 3A.
(XLSX)

S5 Table RNA-Seq data comparing vehicle treated PIAS1KO and ntg cells. (A) RNA-Seq analysis of [PIAS1KO_Veh/ntg_Veh]. (B) Protein coding mRNA from A. (C) Protein coding mRNA with padj<0.05 from B. (D) Protein coding mRNA from C with Log_2_[PIAS1KO_Veh/ntg_Veh] > 2. (E) Reactome analysis of genes in D, pathways highlighted in blue are represented in Fig 4C. This table also provides the data for Fig 4D.
(XLSX)

S6 Table RNA-Seq data comparing TNFα treated PIAS1KO and ntg cells. (A) RNA-Seq analysis of [PIAS1KO_TNFα/ntg_TNFα]. (B) Protein coding mRNA from A. (C) Protein coding mRNA with padj<0.05 from B. This table provides the data for Fig 4G.
(XLSX)

S7 Table Reactome analysis of RNA-Seq data comparing TNFα treated PIAS1KO and ntg cells. Genes significantly enriched in [PIAS1KO_TNFα/ntg_TNFα] from S6 Table (padj<0.05 and Log2FoldChange>2). Gene list (left) imported into reactome analysis tool, reactome report (right). Reactome pathways highlighted in yellow are represented in Fig 4E.
(XLSX)

S8 Table Analysis of RNA-Seq data from GSE137065, cells infected with HCMV or ΔUL26 mutant. (A) Genes depleted in cells infected with HCMV relative to ΔUL26 mutant virus, provides data for Fig 4E, 4F, and 4H. (B) Genes significantly enriched in cells infected with ΔUL26 (Log_2_[HCMV/ΔUL26] < -2 & padj < 0.05). This gene list is the input for reactome analysis. (C) Reactome analysis report of B. Intersecting Reactome pathways from top 10 Reactome terms of [PIAS1KO+TNFα/ntg+TNFα] from S7 Table also significantly perturbed by the absence of UL26 are represented in yellow. The -log10(entities FDR) of these intersecting reactome pathways are represented in Fig 4E.
(XLSX)

S9 Table Identifying the genes enriched in the absence of UL26 or PIAS1. The intersection of entities found in Reactome terms shared by [PIAS1KO+TNFα/ntg+TNFα] from S7 Table and [HCMV/ΔUL26] from S8 Table. Data for Fig 4E–4H.
(XLSX)

S10 Table Primers. Primers used for cloning, BAC recombineering, sequencing, and RT-qPCR.
(XLSX)

S1 Data Minimal datasets. Data to reproduce figure graphs and plots for this manuscript. Each sheet is labeled with the corresponding figure panel to which the data within pertains to.
(XLSX)
