# Supplementary figures and images for: A UL26-PIAS1 complex antagonizes anti-viral gene expression during Human Cytomegalovirus infection
Source: PLoS Pathog. 2024 May 20;20(5):e1012058. doi: 10.1371/journal.ppat.1012058 (PMC11142722; doi:10.1371/journal.ppat.1012058)

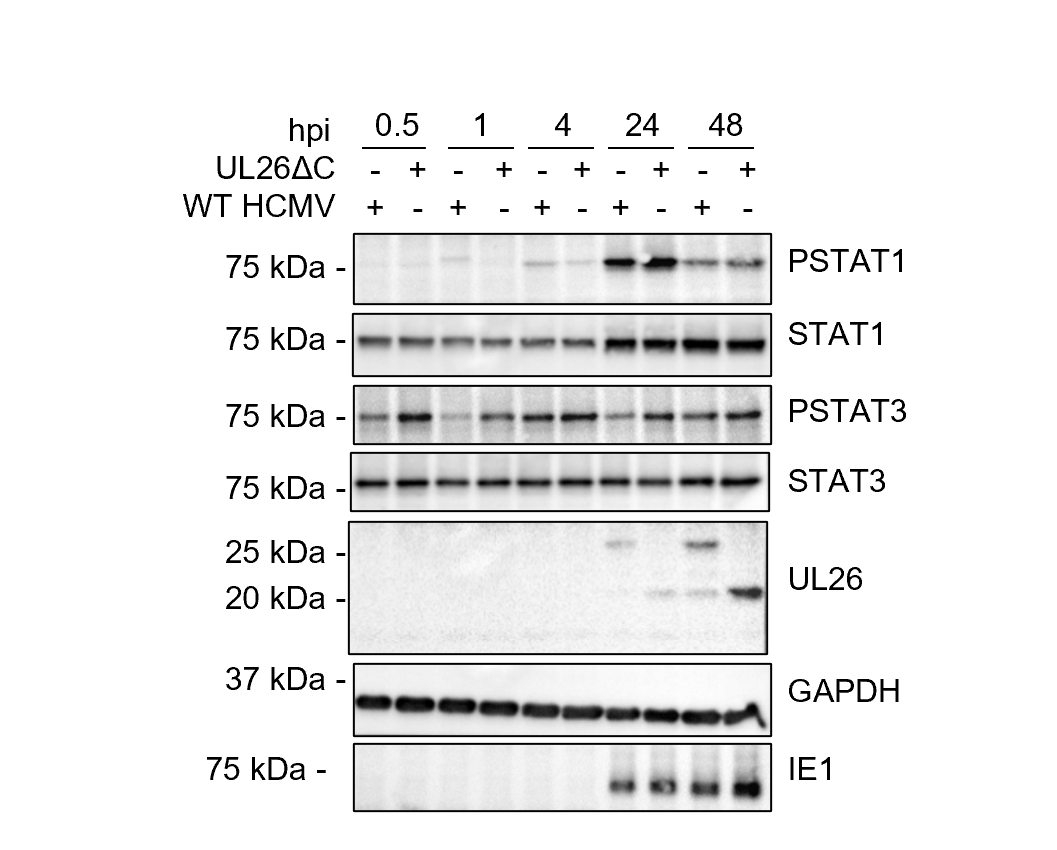

Supplement: S1 Fig — MRC5 cells infected with WT HCMV or UL26ΔC (MOI = 3). Protein harvested at the indicated hour post infection (hpi) and processed by western blot as indicated. (TIF) [file ppat.1012058.s001.tif]

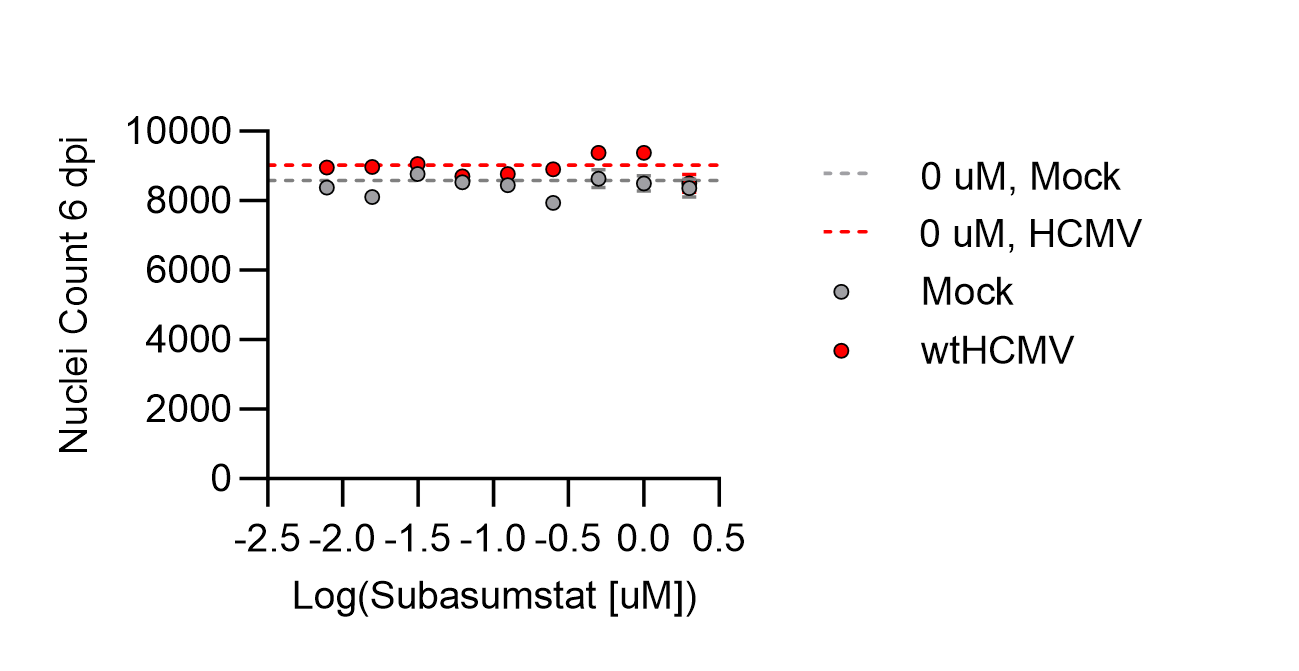

Supplement: S2 Fig — Nuclei count of MRC5 cells infected with Mock (grey) or HCMV that expressed GFP (MOI = 0.05, red) and treated with a 10-point dose curve of Subasumstat at 6 days post treatment (n = 6). Nuclei count of control wells (0 uM, Mock or HCMV) are represented as horizontal dashed lines. (TIF) [file ppat.1012058.s002.tif]

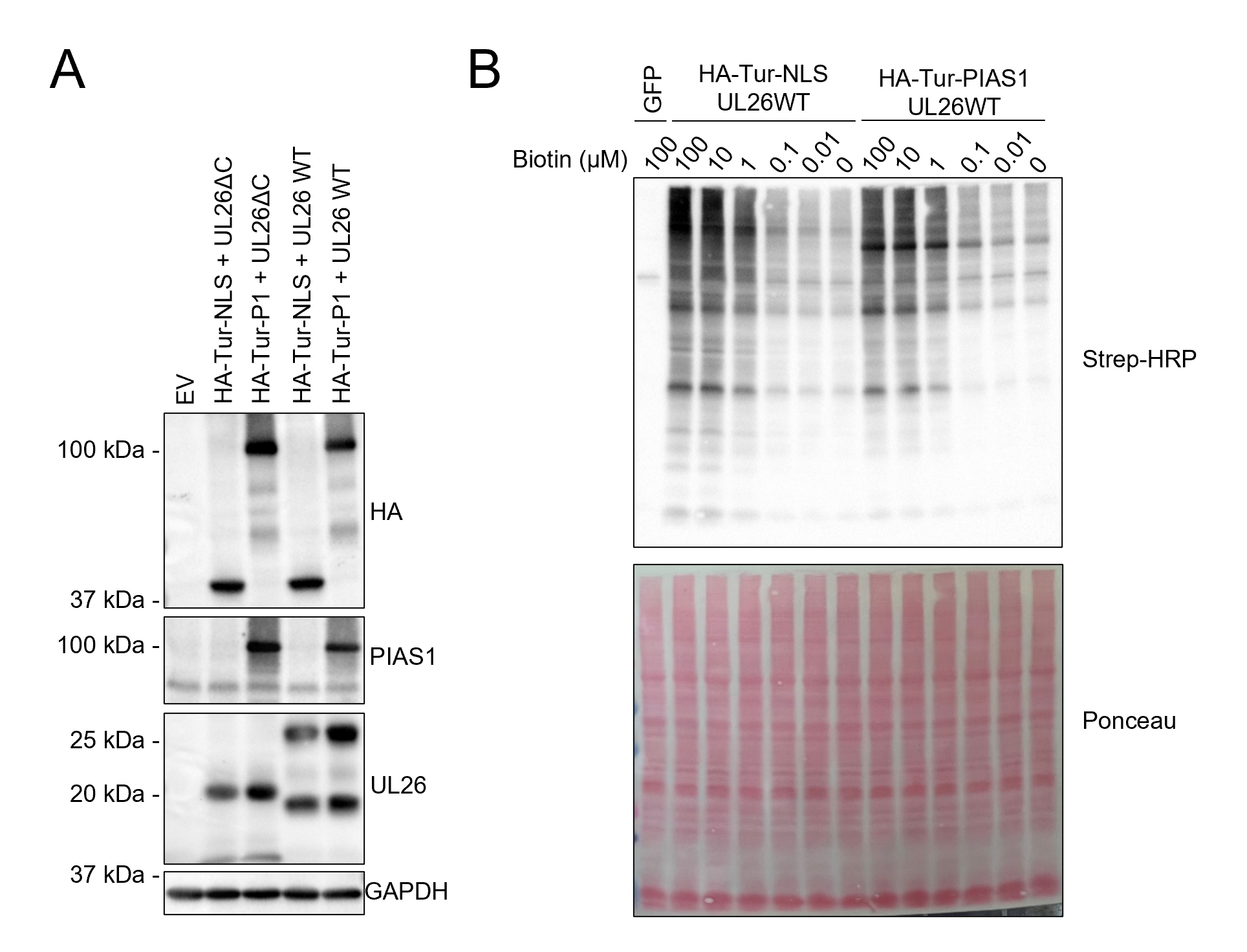

Supplement: S3 Fig — (A-B) HEK293T cells transfected with HA-TurboID-PIAS1 (HA-Tur-P1), HA-TurboID-NLS (HA-Tur-NLS), UL26 WT, UL26ΔC, or empty vector (EV) as indicated. At 47 hours post transfection, cells were treated with the indicated concentration of Biotin. Protein harvested from cells at 48 hours post transfection (1 hr biotin incubation) and processed by western blotting. (A) Expression of TurboID and UL26 proteins. (B) Total protein biotinylation (top) Ponceau stain (bottom). (TIF) [file ppat.1012058.s003.tif]

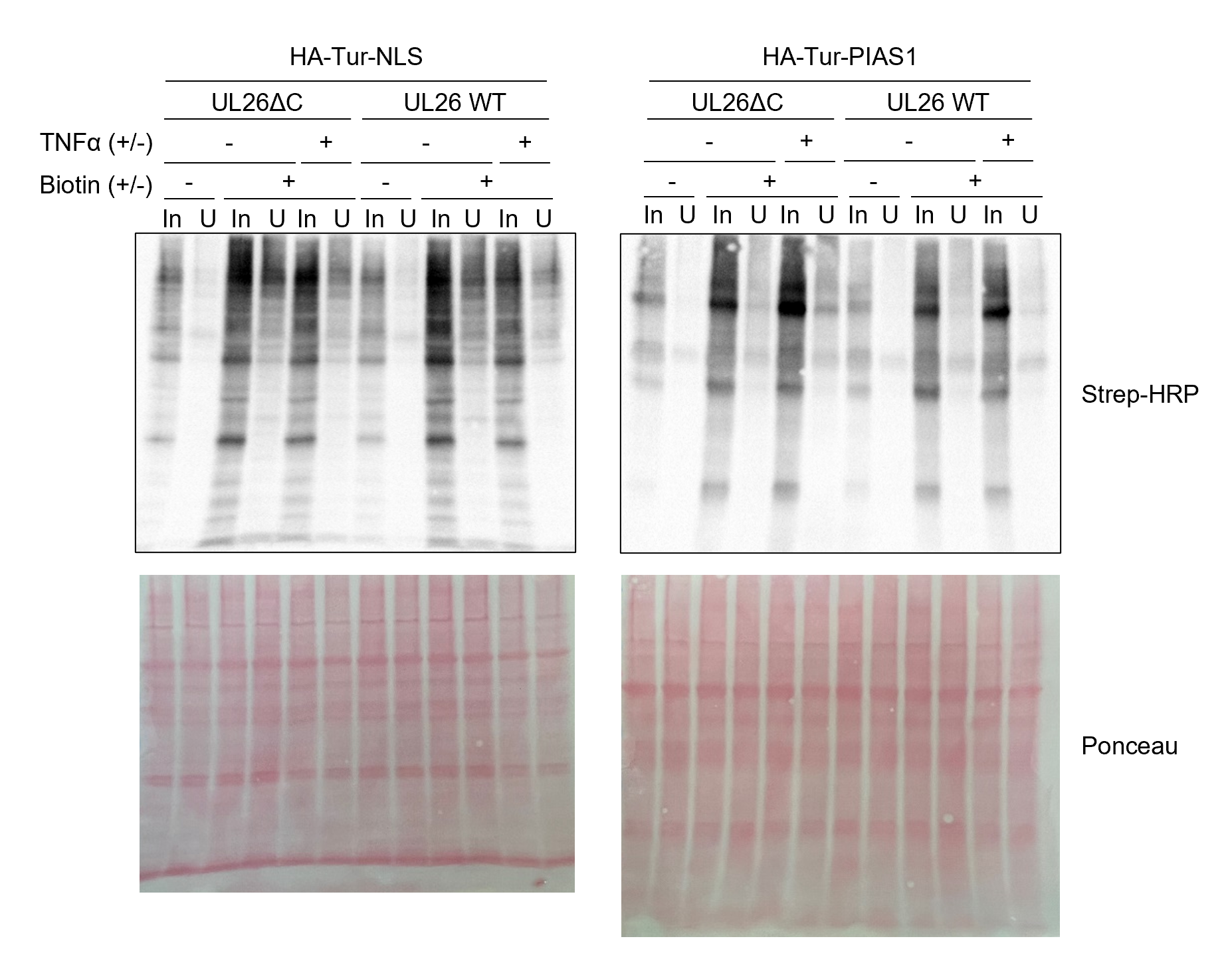

Supplement: S4 Fig — HEK293T cells were transfected with HA-TurboID-PIAS1 (HA-Tur-PIAS1), HA-TurboID-NLS (HA-Tur-NLS), UL26 WT, or UL26ΔC as indicated. At 24 hours post transfection, cells were treated with vehicle or 50 ng/mL TNFα. At 47 hours post transfection, cells were treated with Biotin (1 μM). Protein harvested from cells at 48 hours post transfection (1 hr biotin incubation) and lysed using RIPA buffer for streptavidin-based affinity purification as indicated in materials & methods. Input samples (In) were collected immediately prior to affinity purification. Unbound samples (U) were collected immediately following incubation with streptavidin-bound magnetic beads. Proteins in input and unbound samples were separated by SDS-PAGE and transferred onto nitrocellulose membranes. Membranes were stained with Ponceau to visualize total protein (bottom), destained with TBST, and processed by western blotting for avidin-peroxidase (top). (TIF) [file ppat.1012058.s004.tif]

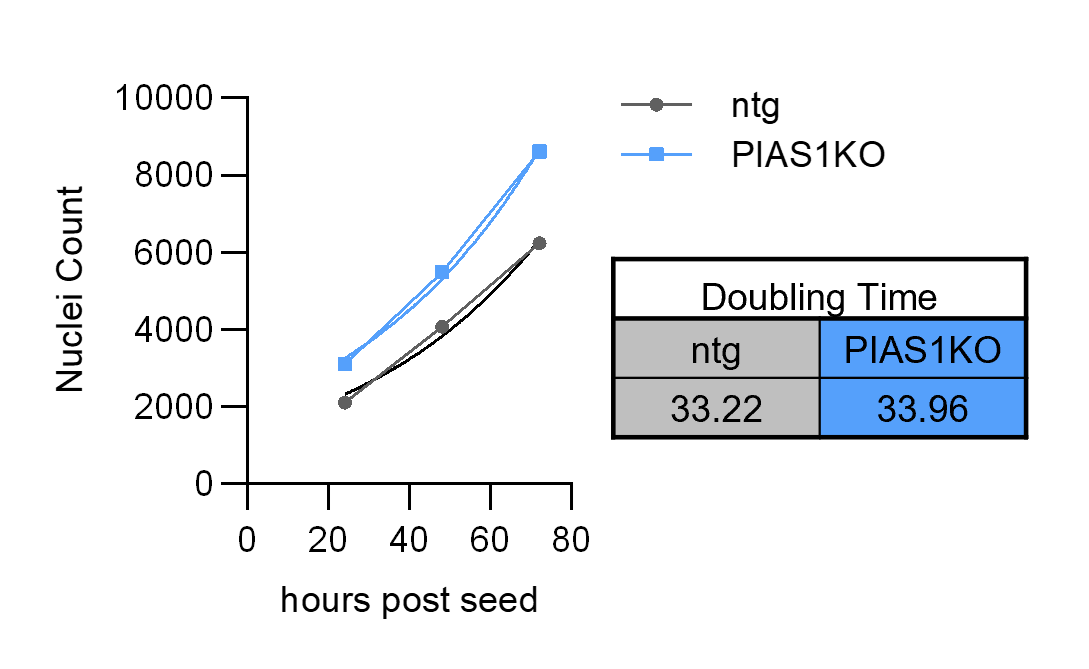

Supplement: S5 Fig — Proliferation rates of MRC5 cells treated with CRISPR RNPs containing non-targeting guide (ntg) or PIAS1 sgRNAs (PIAS1KO). Doubling time (hours) calculated as indicated in materials & methods. (TIF) [file ppat.1012058.s005.tif]

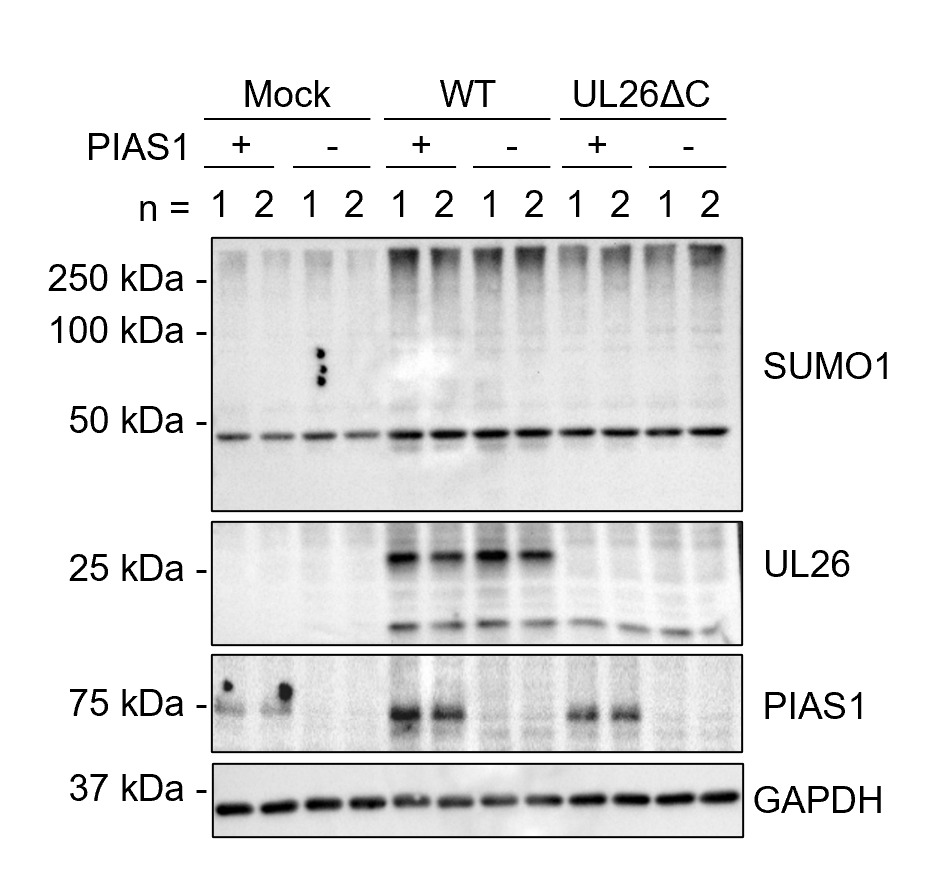

Supplement: S6 Fig — Ntg and PIAS1KO cells infected with Mock, WT HCMV (WT), or UL26ΔC (MOI = 3, n = 2). Protein harvested at 48 hpi and processed by Western analysis as indicated. (TIF) [file ppat.1012058.s006.tif]

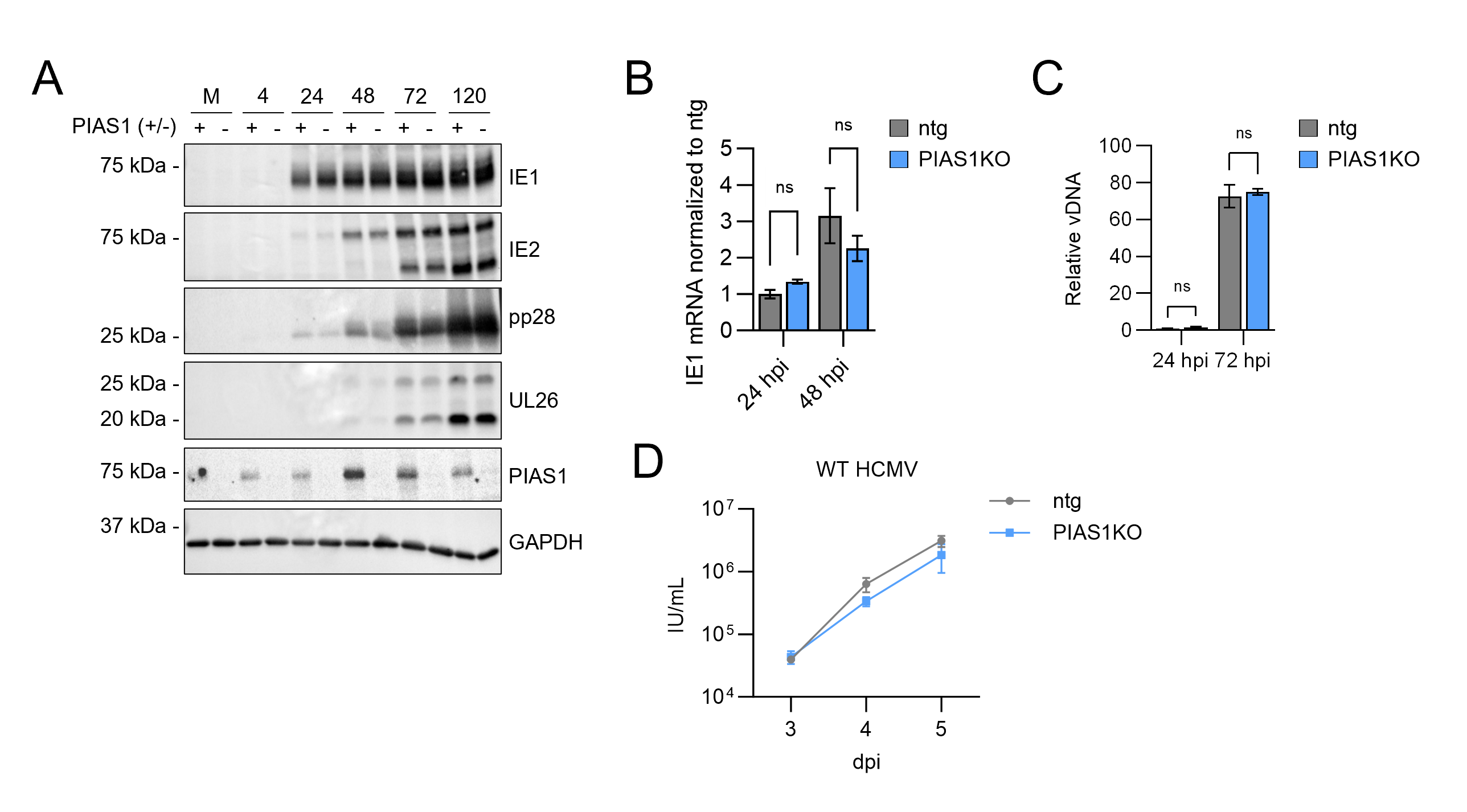

Supplement: S7 Fig — (A-C) Ntg and PIAS1KO MRC5 cells infected with WT HCMV (MOI = 3). Protein (A, n = 1), mRNA (B, n = 3), viral DNA (C, n = 3) was collected and processed for Western analysis (A) or RT-qPCR (B & C). (D) Ntg and PIAS1KO MRC5 cells infected with GFP-expressing WT HCMV (MOI = 3). Virus-containing media samples were harvested at the indicated times post infection and titered in MRC5 cells, titers are represented as IU/mL (D). (B & C) FDR-adjusted p-values determined using 2-way ANOVA followed by two-stage step-up method of Benjamini, Krieger and Yekutieli n.s = not significant, *p<0.033, **p<0.002, ***p<0.001. (TIF) [file ppat.1012058.s007.tif]
